# Prototissues: Assembly strategies, collective behaviors, and emerging applications

**DOI:** 10.1016/j.bioactmat.2025.12.033

**Published:** 2026-01-10

**Authors:** Ziqi Liu, Yiming Wang, Wei Pei, Yi-Xin Huo, Yuan Lu

**Affiliations:** a Key Laboratory of Molecular Medicine and Biotherapy, Aerospace Center Hospital, School of Life Science, Beijing Institute of Technology, Beijing, 100081, China; b State Key Laboratory of Green Biomanufacturing, Department of Chemical Engineering, Tsinghua University, Beijing, 100084, China; c Key Laboratory of Industrial Biocatalysis, Ministry of Education, Department of Chemical Engineering, Tsinghua University, Beijing, 100084, China; d Tangshan Research Institute, Beijing Institute of Technology, Tangshan, 063000, Hebei, China

**Keywords:** Protocell, Prototissue, Collective behavior, Signal communication

## Abstract

Recent advances in bottom-up synthetic biology have significantly expanded the ability to construct artificial life systems. While most efforts focus on building protocells, many biomimetic functions arise only when multiple units operate collectively. Prototissues, formed from interconnected protocell assemblies, provide a platform for such emergent behaviors and offer broad potential in biomedicine, biosensing, and smart materials. This review introduces a dual-dimensional framework for understanding prototissue design. The first dimension examines inter-protocell adhesion strategies that define molecular connectivity, and the second examines spatial programming approaches that organize protocells into functional architectures. On this basis, the review summarizes key collective behaviors enabled by these design principles and highlights how advances in materials chemistry, synthetic biology, and advanced manufacturing support the development of increasingly adaptive and functional prototissues. Major challenges remain, including achieving dynamic and selective adhesion, scaling spatial architectures while maintaining resolution, improving signal transport, and enhancing biological integration. The review outlines potential pathways to address these issues and to guide the development of prototissues with more sophisticated, life-like properties. Overall, the conceptual framework and insights presented here provide a foundation for the rational design of next-generation prototissues and advance bottom-up synthetic biology toward more complex artificial life systems.

## Highlights

• Prototissues are synergistic protocells with advanced biomimetic functions.
• They can achieve innovative solutions in science, biomedicine, and materials.
• Collective intelligence technologies drive state-of-the-art development.
• Existing challenges and future outlooks are carefully analyzed and summarized.
• The insights and frameworks would advance more complex artificial life systems.

## Graphical abstract

**Figure:**
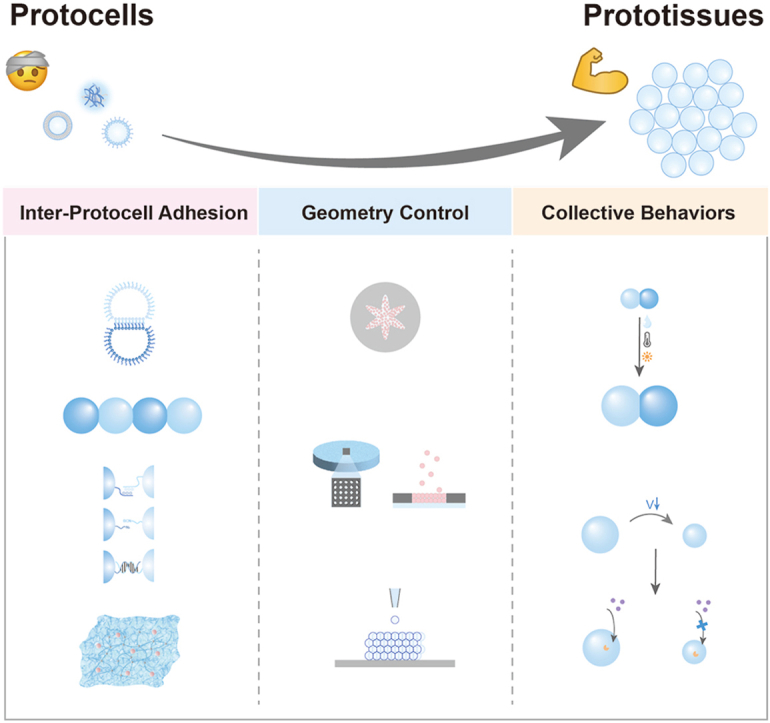

## 1 Introduction

Over a century ago, John Tyndall remarked that evolutionary doctrine, despite explaining the progression of life, “gives us no hint as to the physical mechanism of such generation … or in what manner differences of environment have acted in order to modify them.” This observation highlights a longstanding gap between recognizing the fact of biological evolution and understanding the physicochemical processes that give rise to life-like behaviors [1]. Modern synthetic biology, particularly its bottom-up branch, is seeking to reconstruct life-like functions using minimal, programmable components [2]. Central to this vision is the development of systems in which molecular interactions, reaction networks, and physical organization can be rationally engineered rather than inherited [3]. Foundational advances in this field have established key principles—modularity, hierarchical design, and composability—that enable biological functions to be deconstructed into controllable units. Protocells emerged as an important platform within this framework, offering simplified micro-compartments that permit experimental interrogation of encapsulation, selective transport, energy transduction, and synthetic signaling pathways under well-defined physicochemical conditions [1]. These reductionist constructs provide valuable opportunities to explore how life-like behaviors arise from non-living components.

During the past decade, protocell research has progressed from isolated micro-compartments to increasingly integrated assemblies [[4], [5], [6], [7], [8]]. Different protocell systems, including lipid vesicles, coacervate droplets, polymer-based proteinosomes, and hybrid structures, address specific challenges related to stability, permeability, or functional incorporation. Lipid-based vesicles mimic biological membranes and allow tunable molecular exchange [9]. Coacervates create dynamic and compositionally enriched microenvironments that support biochemical reactions [8,10]. Polymeric vesicles offer improved mechanical robustness [11]. Hybrid systems combine complementary advantages from multiple material classes [12].Despite these advances, single protocells still fall short in mimicking the architectural complexity and emergent behaviors observed in multicellular systems. In natural tissues, functions such as force coordination, pattern formation, compartmentalized communication, and distributed decision-making emerge from collective organization rather than isolated units [13,14].This limitation has motivated growing interest in synthetic assemblies that are capable of coordinated, tissue-like behaviors.

These developments have led to the concept of prototissues. Prototissues are multicompartment assemblies that emulate essential features of natural tissues through spatial organization and programmable interactions between units [[15], [16], [17]]. Biological tissues depend on hierarchical architecture [18], finely regulated cell–cell adhesion [19,20], selective communication through chemical, ionic, and mechanical pathways [21], and dynamic coupling to the extracellular matrix [22]. These factors work together to coordinate group-level behaviors that cannot be produced by individual cells. Prototissues aim to recreate these principles within experimentally accessible systems. They support studies of emergent phenomena such as collective sensing, integrated signal processing, mechanical adaptation, and metabolic cooperation. In this way, prototissues function both as bioinspired materials and as model systems for examining multicellular organization. Their potential relevance to biomedical research continues to grow, especially in areas such as microtissue modeling, disease mechanism studies, and therapeutic delivery [17,[23], [24], [25], [26]].

Engineering such systems raises fundamental questions that this review seeks to consolidate: (1) How do different adhesion mechanisms govern the connectivity and collective dynamics of protocell assemblies? (2) How do spatial organization strategies—from templated assembly to field-directed and micromanipulation-based methods—shape emergent structure–function relationships? (3) How do prototissues respond to mechanical, chemical, or thermal cues, and what principles underlie their adaptive or mechanochemical behaviors? (4) How do engineered assemblies exchange information through molecular, ionic, and electrical pathways, and how can these mechanisms be leveraged for functional applications? (5) What cutting-edge technologies—manufacturing, intelligent materials, synthetic biology tools, and computational approaches—are expanding the design space for future prototissues? (6) Finally, what opportunities and challenges define their emerging applications in biology, medicine, and engineering?

**Fig. 1:**
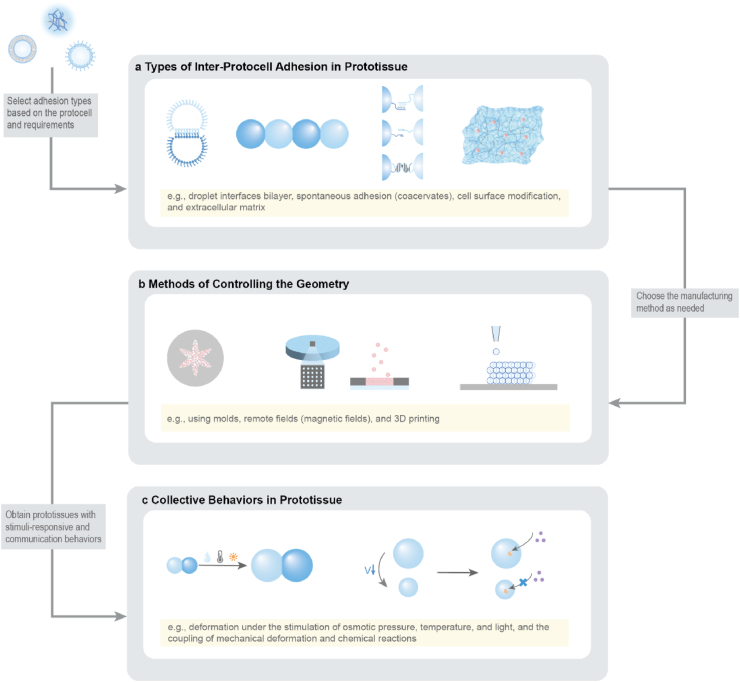
Schematic illustration of the dual-dimensional synergistic framework for prototissue engineering and its functionalities. (a) Molecular-scale adhesion engineering strategies achieve programmable inter-protocell connectivity by designing surface interactions (e.g., biomolecular recognition, covalent bonding, electrostatic interactions). (b) Macroscale spatial programming strategies organize protocells into defined geometric architectures using physical fields, templates, or printing techniques. (c) Emergent collective behaviors arising from the synergistic framework, unattainable by individual protocells, including stimuli-responsive mechanics, mechanochemical coupling, and multimodal signal communication.

To provide a coherent framework for understanding how prototissues are constructed and how their collective behaviors arise, this review organizes current progress around a dual-dimensional perspective (Fig. 1a–b): (i) molecular adhesion strategies that define inter-unit connectivity, and (ii) spatial programming methods that shape emergent architecture and function. The discussion further considers how prototissues respond to external cues (Fig. 1c), how they enable chemical and electrical communication, and how advances in manufacturing technologies, responsive materials, synthetic biology, and computational design support increasingly sophisticated collective behaviors. Finally, emerging opportunities across fundamental biology, biomedical engineering, and adaptive bionic technologies are outlined, emphasizing the role of prototissues as platforms for mechanistic inquiry and as foundations for future bioinspired functional systems.

## 2 Mechanisms of inter-protocell adhesion in prototissue assembly

Intercellular adhesion is the architectural foundation of biological tissues, supporting structural integrity and coordinated functions such as mechanical force transmission and signal communication [14,[27], [28], [29]]. To replicate these principles in synthetic prototissues, three complementary adhesion paradigms have been developed To translate these biological principles into synthetic prototissues, three complementary adhesion paradigms have been developed (Table 1). These include direct adhesion based on intrinsic protocell physicochemical properties, engineered molecular adhesion through designed electrostatic, covalent, or biomolecular recognition, and extracellular matrix (ECM) -mediated adhesion that mimics indirect cell–matrix coordination within synthetic scaffolds.

**Table 1:** Summary of adhesion paradigms used in prototissue assembly.

Adhesion paradigm	Principle	Control factors	Advantages	Limitations
**Direct adhesion**	Ca^2+^-induced hemifusion [[30], [31], [32], [33]], NaCl-driven vesicle interfacial membranes (VIMs) [34],droplet interface bilayers (DIBs) [[35], [36], [37], [38], [39], [40], [41], [42], [43], [44], [45], [46], [47], [48], [49], [50], [51]],coacervate/coacervate interfacial tension-driven adhesion [52,[53], [54]]	Ion concentration (Ca^2+^, NaCl), oil-phase environment (for DIBs), interfacial tension	Simple formulation, minimal engineering requirements, stable or semi-stable junction formation, support for collective behaviors such as enzyme cascades and mechanical coupling	Irreversibility of hemifusion, limited specificity in some modes, low permeability of VIMs, requirement of an oil-phase environment for DIBs
**Engineered adhesion – electrostatic**	Polymer-mediated charge bridging [55,56], complementary surface charge interactions [57,58], genetically encoded charged motifs [59]	pH, ionic strength, polymer architecture, density of charged groups, genetically encoded peptide charges	Reversibility or semi-reversibility, simple formulation, environmental tunability, suitability for basic dynamic assembly	Limited specificity, moderate binding strength, narrow pH tolerance, reduced orthogonality due to exogenous polymers
**Engineered adhesion – covalent functional groups**	Azide–alkyne click reactions [[60], [61], [62], [63], [64]], disulfide exchange [61], hydrazone formation [61], PBA–diol formation [65]	Chemical functionalization density, redox state, presence of competing ligands, light or pH triggers for dynamic covalent bonds	High mechanical robustness, strong and long-term stability, chemical programmability, compatibility with large or hierarchical architectures	Limited reversibility in many systems, reduced adaptability from covalent coupling, constraints imposed by membrane compatibility of reactions
**Engineered adhesion – biomolecular recognition**	Biotin–streptavidin binding [29,66], lectin–glycan interactions [67,68], optogenetic receptor pairs [[69], [70], [71], [72]], DNA hybridization [[73], [74], [75], [76], [77]]	Ligand density, glycan pattern, light input, hybridization temperature	High specificity, high programmability, reversible and spatiotemporally controllable interactions, capacity for logic-like behaviors and selective assembly	Near-irreversibility of pairs such as biotin–streptavidin, ion sensitivity of hybridization, dependence of optogenetic systems on illumination equipment, higher cost and design complexity
**ECM-mediated adhesion**	Matrix-mediated contact between protocells within hydrogel scaffolds [35,[46], [47],[78], [79], [80], [81], [82]]	Hydrogel composition, crosslinking density, porosity, ionic environment	Large-scale spatial organization, reaction–diffusion signaling capability, tissue-like mechanical context, compatibility with biological pathways	Spatial resolution limited by matrix porosity, slower assembly dynamics, difficulty of reconfiguration after embedding

### 2.1 Direct protocell adhesion

**Fig. 2:**
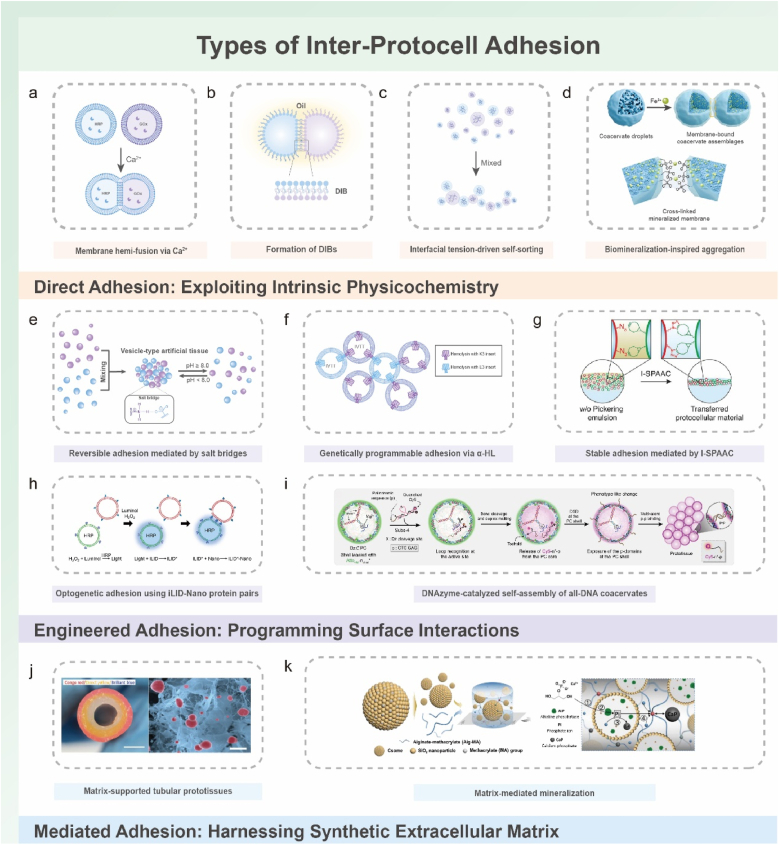
Inter-protocell adhesion mechanisms and representative strategies. (a–d) Direct adhesion exploiting intrinsic physicochemical interactions. (a) Ca^2+^-induced hemifusion between lipid vesicles, where divalent ions screen electrostatic repulsion and drive the formation of metastable stalks that generate hemi-fused junctions. Reproduced with permission [30]. Copyright 2019, Royal Society of Chemistry. (b) Droplet interface bilayers (DIBs) formed as lipid-coated aqueous droplets in oil create planar bilayers at points of contact. Adapted with permission [39]. Copyright 2025, Wiley-VCH. (c) Interfacial-tension-driven self-sorting of coacervate droplets with distinct interfacial tensions, producing ordered linear or branched arrangements. Reproduced with permission [52]. Copyright 2025, Wiley-VCH. (d) Biomineralization-inspired aggregation, in which Fe^3+^/pH-regulated mineral shells around coacervates modulate adhesion and enable reversible assembly through Fe^2+^/Fe^3+^ redox cycling. Reproduced with permission [83]. Copyright 2025, Wiley-VCH. (e–i) Engineered adhesion via programmed surface interactions. (e) pH-responsive salt-bridge adhesion between amphoteric vesicle surfaces, enabling reversible assembly and disassembly. Adapted with permission [57]. Copyright 2024, Wiley-VCH. (f) Genetically programmable electrostatic adhesion using engineered α-hemolysin (α-HL) pores bearing complementary K3/E3 charged peptide loops that mediate selective bridging. Adapted with permission [59]. Copyright 2024, Springer Nature. (g) Bioorthogonal covalent adhesion via interfacial strain-promoted azide–alkyne cycloaddition (I-SPAAC), producing stable interfacial junctions in proteinosomes or emulsions under catalyst-free aqueous conditions. Reproduced with permission [62]. Copyright 2021, Wiley-VCH. (h) Optogenetic adhesion using iLID–Nano light-responsive protein pairs to achieve reversible blue-light-activated binding between protocells. Reproduced with permission [71]. Copyright 2023, American Chemical Society. (i) DNAzyme-catalyzed self-assembly of all-DNA coacervates, where catalytic substrate cleavage releases palindromic sequences that drive multivalent intermolecular adhesion. Reproduced with permission [74]. Copyright 2022, Springer Nature. (j–k) Matrix-mediated adhesion and ECM-inspired stabilization. (j) Matrix-supported tubular prototissues formed by embedding enzyme-loaded coacervate vesicles within hydrogel frameworks that stabilize multi-compartment assemblies and support reaction–diffusion coupling. Reproduced with permission [80]. Copyright 2022, Springer Nature. (k) Matrix-mediated mineralization, in which enzyme-functionalized colloidosomes or protocells within hydrogels direct spatially patterned inorganic deposition, generating stable protocell–matrix cohesion. Reproduced with permission [82]. Copyright 2025, Springer Nature.

Direct adhesion strategies exploit the innate physicochemical properties of protocell boundaries such as lipid membrane thermodynamics and coacervate interfacial tension. These intrinsic mechanisms orchestrate connectivity without requiring engineered surface ligands (Fig. 2a). This minimalist paradigm responds to environmental cues, including ionic strength, temperature, and pH, which modulate adhesion dynamics.

#### 2.1.1 Liposomes networks: membrane-mediated direct adhesion

Lipid-based protocells, including giant unilamellar vesicles (GUVs) and lipid-coated droplets, achieve cohesion through environmentally tuned membrane interactions while avoiding the need for exogenous crosslinkers. Three principal mechanisms define this category, each with characteristic trade-offs in permeability, reversibility, and stability: Ca^2+^-induced hemifusion [30,31,32], NaCl-modulated vesicle interfacial membranes (VIMs) [34], and droplet interface bilayers (DIBs) [[35], [36], [37], [38], [39], [40],^42^,^43^,[45], [46], [47], [48], [49], [50], [51],^84^].

Ca^2+^-induced vesicle hemifusion begins with cation-mediated bridging of anionic lipid headgroups, which screens electrostatic repulsion and drives the formation of metastable stalk intermediates, ultimately producing a micellar fusion pore (Fig. 2a). This configuration yields a contiguous aqueous conduit that supports rapid molecular exchange, such as H_2_O_2_ diffusion, and can increase resorufin production rates at fusion sites by 2–3 fold compared with unfused controls [30]. The irreversible nature of hemifusion provides strong mechanical cohesion and enables the construction of freestanding prototissues capable of substrate detachment for functions including sustained nitric oxide (NO) release [31]. The structural robustness of hemifused junctions also underpins synchronized collective behaviors; linear GUV colonies interconnected by hemifusion display ATP-driven contractile rhythms that resemble muscle-like coordination because the permanent linkages prevent desynchronization during cyclic deformation [32].

In contrast, NaCl-modulated VIMs provide a reversible adhesion strategy. Electrostatic screening by NaCl promotes the close apposition of two intact lipid bilayers without lipid mixing, resulting in a dual-bilayer junction [34]. This configuration supports cyclic assembly and disassembly of vesicle networks in response to ionic strength. However, the dual-bilayer structure imposes a substantial diffusional barrier that can limit intervesicular communication. Functional reconstitution of transmembrane protein pores into VIMs has been used to mitigate this limitation, enabling selective molecular transport such as Ca^2+^ flux and linking reversible structural connectivity with more controlled signaling behaviors.

DIBs represent a thermodynamically distinct approach in which lipid monolayers at oil–water interfaces assemble into bilayers between adjacent aqueous droplets (Fig. 2b) [85]. This process is reversible and yields structurally stable junctions, positioning DIBs as a useful platform for reconfigurable networks and studies of controlled molecular transport. Early work employed 3D printing to generate DIB-based networks that showed osmotically driven collective shape changes [50]. A longstanding limitation of conventional DIBs is their confinement within a bulk oil phase, which restricts communication with the surrounding aqueous environment. This limitation has been addressed by engineering water-in-oil-in-water (W/O/W) double emulsions, where capillary forces guide the assembly of GUVs that form DIBs interfacing with both neighboring droplets and the external aqueous phase [40]. This architectural advance enables bidirectional molecular exchange and expands potential applications in biosensing and in models of cell–environment communication relevant to biomedical testing.

In summary, membrane-mediated adhesion spans a functional continuum. Ca^2+^-induced hemifusion provides high permeability and mechanical robustness but is irreversible. VIMs support reversible assembly yet require engineering interventions to address inherent diffusional barriers. DIBs enable stable and reversible bilayer formation with notable versatility, and recent developments have mitigated their historical isolation from aqueous environments. Together, these mechanisms highlight how membrane physics alone can achieve tunable cohesion, with implications for constructing biocompatible prototissues suited for therapeutic delivery and microphysiological modeling.

#### 2.1.2 Coacervate droplet networks: programmable aggregation

Coacervate droplets, formed via liquid–liquid phase separation (LLPS), serve as compelling protocell models because they can selectively sequester biomolecules and create biomimetic crowded microenvironments. Prototissue assembly strategies based on coacervates broadly fall into two categories: membrane-free droplets and membranized systems, with the latter providing enhanced stability and functional sophistication. A hallmark of coacervate-based prototissues is their pronounced dynamic responsiveness to a spectrum of environmental stimuli, including light, ionic strength, pH, and redox potential, often exceeding the adaptive range of purely lipid-based systems [8]. This high degree of responsiveness is particularly attractive for designing prototissues that interface with fluctuating biological environments, for example in controlled release and dynamic regulation of bioactive factors.

Membrane-free coacervates typically self-organize through interfacial tension differences arising from their intrinsic physicochemical compositions. For instance, differential interfacial tensions between liquid-crystalline and homogeneous coacervate phases (e.g., DDAB/trans-AzoAsp_2_ and PDDA/trans-AzoAsp_2_) can drive spontaneous self-sorting into architecturally defined linear or branched networks (Fig. 2c) [54]. The resulting hemispherical contact junctions permit spatial compartmentalization of biomolecules and enable localized enzymatic catalysis. The structural reconfigurability of these networks, often mediated by host–guest interactions or photoisomerization, reflects a primitive form of stimulus-responsive tissue plasticity. A complementary strategy generates hybrid networks from associative LLPS coacervates (DEAE-dex/PASP) and segregative aqueous two-phase system (ATPS, dextran/PEG) droplets. Interfacial tension equilibria between these phases induce partial engulfment, yielding worm-like chains with alternating coacervate and dextran domains [52]. This design supports macroscopic biomolecular sorting and regulation of enzyme cascade kinetics, where environmental perturbations (e.g., altered salt concentration) can selectively dissociate specific phases to modulate reaction fluxes. Additionally, studies on compartmentalized biomolecular condensates have shown that nucleation can be dynamically regulated by temperature and salt concentration to generate multiphase coacervates with distinct dense phases [8,86]. Such strategies provide a conceptual and practical basis for prototissues with advanced spatial organization and controllable reaction environments that are relevant to biochemical and therapeutic modulation.

Despite their dynamic assembly, membrane-free coacervates are often limited by inherent thermodynamic instability and restricted functional complexity. Membranized coacervate systems have therefore been developed to address these shortcomings. For example, coating coacervates with metal–organic framework (MOF) nanoparticles enhances stability and introduces molecular sieving properties. Subsequent electrolyte-induced (e.g., NaCl) shielding of electrostatic repulsion allows these membranized protocells to pack into dense, tissue-like architectures [87]. A further advance is a bioinspired strategy that directly couples membranization and aggregation (Fig. 2d) [83]. By precisely modulating Fe^3+^ concentration and pH, the thickness of a mineralized shell around coacervates can be controlled, thereby dictating their aggregation state. The Fe^2+^/Fe^3+^ redox cycle confers dynamic reversibility to both membrane formation and macroscopic network assembly and disassembly, establishing a versatile chemical platform for constructing life-like adaptive systems that could, in principle, respond to pathophysiologically relevant redox environments.

Overall, direct adhesion offers operational simplicity and dynamic environmental responsiveness, exemplified by light-triggered coacervate reconfiguration. However, inherent limitations persist, including structural instability under physiologically relevant conditions (e.g., DIBs’ hydrophobic confinement), restricted molecular diffusion across multi-bilayer junctions, and limited binding specificity. These constraints highlight that engineered adhesion strategies are required to achieve higher-order robustness, selectivity, and long-term functionality, particularly in prototissues intended for therapeutic delivery, regenerative constructs, or microphysiological models.

### 2.2 Surface electrostatic interactions-mediated adhesion

While direct adhesion strategies often leverage electrostatic forces as secondary effects of environmental conditioning (e.g., ionic screening), this section highlights engineered approaches in which electrostatic interactions serve as the primary, intentionally designed driving force for prototissue assembly. Electrostatic adhesion provides a versatile paradigm by exploiting complementary surface charges, with binding affinity and specificity tunable through environmental parameters such as pH and ionic strength. The development of these strategies indicates a progression from externally mediated bridging toward increasingly autonomous and bio-integrated systems, spanning (i) polyelectrolyte bridging, (ii) environmentally responsive salt bridges, and (iii) self-encoded adhesion.

The most straightforward approach involves polyelectrolyte bridging, in which cationic biopolymers mediate the assembly of like-charged anionic GUVs. For example, poly-L-arginine (PLA) induces localized charge inversion on vesicle surfaces, overcoming electrostatic repulsion and forming stable clusters through “mediated-attraction” junctions [56]. Similarly, oleate-functionalized anionic GUVs can be crosslinked through PLA bridges, achieving moderate adhesion energies [55]. Although effective for proof-of-concept assembly, this exogenous bridging strategy presents notable limitations. The manual introduction of polymers restricts scalability and spatiotemporal control, and non-specific interactions may compromise the mechanical robustness and functional fidelity of the resulting prototissues, limiting their suitability for biomedical applications requiring dynamic reorganization and controlled bioactive release.

To address these constraints, research has shifted toward intrinsic control mechanisms that endow protocells with environmental responsiveness [57,58]. Systems incorporating zwitterionic membrane additives (e.g., amines, carboxylic acids) exemplify this approach, autonomously driving vesicle adhesion through pH-tunable electrostatic and hydrogen bonding (Fig. 2e) [57]. These engineered “salt bridges” exhibit high reversibility, maintaining tissue-like architectures within a narrow physiological pH window (e.g., pH 7.2–7.8) and rapidly disassembling upon slight increases in pH (e.g., to pH 8.0) as intermolecular bonding weakens. This shift toward autonomous behavior moves beyond passive material responses and introduces a primitive form of environmental sensing and feedback. However, such physicochemical switches, although reversible, still rely on relatively nonspecific interactions and lack the molecular fidelity characteristic of biological adhesion systems.

A transformative advancement arises from integrating synthetic biology with protocell design, establishing the paradigm of self-encoded electrostatic adhesion. In a landmark study, Harjung et al. introduced genetically specified charged peptide loops (K3/E3) into α-hemolysin (α-HL) pores, generating programmable salt bridges between adjacent protocells (Fig. 2f) [59]. This biomimetic strategy yields two major advances. First, it recapitulates aspects of natural membrane functionalization by leveraging genetic pathways for protein expression and localization, linking adhesion capability to protocell state. Second, it removes the need for external triggers or manual intervention, enabling autonomous and specific adhesion based on the genetically programmed phenotype of the constituent protocells. The α-HL pores also serve a dual function, mediating adhesion while facilitating exchange of small-molecule signals, which enhances intra-tissue communication. This work represents a conceptual transition from passive material responses toward adaptive, life-like interfacial engineering, where adhesion emerges from the protocell's synthetic genome.

In summary, electrostatic strategies for prototissue assembly have progressed from simple, externally controlled bridging to increasingly autonomous systems. The shift from polyelectrolyte mediators to environmentally responsive membranes, and ultimately to genetically encoded adhesion motifs, illustrates a growing integration between materials science and synthetic biology. This direction suggests a future in which prototissues can self-assemble and dynamically reconfigure through programmed genetic circuits and internal states, thereby more closely approximating the adaptive behaviors of living tissues.

### 2.3 Surface functional group-mediated adhesion

The dynamic, reversible nature of electrostatic adhesion, while advantageous for certain adaptive behaviors, is intrinsically limited by its relatively low binding strength and specificity. These constraints become critical in applications requiring long-term structural integrity under physiological stress or precise spatial patterning. To address these challenges, surface functional group–mediated strategies have been developed that leverage the robust and programmable connectivity of covalent chemistry. This paradigm uses tailored chemical modifications on protocell membranes to create inter-protocell linkages with defined kinetics and stability, offering a route toward more durable and biointegration-ready prototissue architectures. The field is broadly divided into two complementary approaches (1) the use of static, irreversible covalent bonds (e.g., click chemistry): for maximum structural stability, and (2) the implementation of dynamic covalent bonds (DCBs) to introduce stimuli-responsive reversibility into the prototissue fabric.

#### 2.3.1 Click chemistry

Click chemistry, pioneered by Sharpless et al., provides a powerful toolkit for bioorthogonal ligation and enables efficient, modular, high-fidelity bonding under mild aqueous conditions—a prerequisite for biocompatible assembly [88]. The foundational copper-catalyzed azide–alkyne cycloaddition (CuAAC) supports the covalent assembly of functionalized polymer vesicles into macroscopic structures. Control over the assembly outcome—from membrane fusion to nonfusogenic adhesion—can be achieved by adjusting the stoichiometry and presentation of reactive groups such as the alkynyl-to-azide ratio [89]. However, the cytotoxicity associated with residual copper catalysts remains a major obstacle to biomedical translation.

This limitation has been addressed by metal-free alternatives, most notably the strain-promoted azide–alkyne cycloaddition (SPAAC). The exceptional bioorthogonality, rapid kinetics, and metal-free nature of SPAAC establish it as a versatile platform for constructing complex biological architectures [90]. Its utility in prototissue engineering is illustrated by the hierarchical assembly of azide- and cyclooctyne-functionalized proteinosomes within W/O/W Pickering emulsions, yielding spheroidal prototissues through interfacial crosslinking [60]. A key feature of these covalently stabilized assemblies is their ability to display emergent collective dynamics; for example, thermally responsive volume changes (up to 45 %) are governed by crosslink density, demonstrating how static covalent bonds can paradoxically regulate dynamic macroscopic behavior. The synergy between SPAAC and advanced manufacturing methods—such as microfluidics for programmable 3D architectures [63] and floating-mold approaches for centimeter-scale reaction–diffusion networks (Fig. 2g) [62,64], further underscores its value in fabricating structurally defined and functionally stable prototissues.

#### 2.3.2 Dynamic covalent bonds

Despite their robustness, the irreversible nature of classic click chemistry limits the adaptability and reconfigurability of the resulting prototissues. Dynamic covalent bonds (DCBs)—including disulfide, hydrazone, and phenylboronic acid (PBA)–diol interactions—offer a potential solution by enabling reversible association–dissociation or exchange reactions in response to environmental cues such as pH, redox potential, light, or competing ligands. These features position DCBs as promising mechanisms for creating self-healing, recyclable, and adaptive prototissue networks [91].

However, translating this considerable theoretical potential into functional and reliable systems has proven challenging, highlighting a persistent gap between chemical feasibility and practical implementation in protocellular environments. For example, while disulfide linkages formed via thiol oxidation can mediate protocell aggregation into stable clusters [61], attempts to induce controlled disassembly using reducing agents often cause nonspecific vesicle rupture rather than preserving membrane integrity, underscoring the difficulty of achieving benign reversibility. Similarly, hydrazone bonds, although chemically reversible under acidic conditions [61], have not been rigorously demonstrated to support reversible assembly–disassembly cycles in physiologically relevant environments, leaving their dissociation kinetics and spatial control largely uncharacterized. Boronate ester–based systems [65], which exploit pH- and temperature-sensitive PBA–diol equilibria [92], have been used to crosslink polysaccharide-coated proteinosomes into functional aggregates; yet achieving on-demand, macroscopic network reconfiguration remains an unmet challenge.

This persistent disconnect underscores several key hurdles in DCB design: chemical reversibility must operate under biocompatible conditions without compromising protocell stability, and the kinetics of bond exchange must align with the timescales required for tissue-level reconfiguration. At present, DCBs provide primarily a conceptual pathway toward adaptive prototissues, with their practical realization dependent on advances in bond design, reaction tuning, and integration with protocell-compatible environments.

To sum up, covalent adhesion strategies play a pivotal role in endowing prototissues with structural resilience. Static click chemistry enables robust, bioorthogonal bonding for precision assembly across scales, from nanoscale hybrids to centimeter-scale networks. In contrast, DCBs offer a promising—though not yet fully realized—route to incorporating life-like adaptability through stimuli-responsive reversibility. Collectively, these functional group–mediated strategies provide superior stability and programmability compared with physical interactions. However, their dependence on synthetic chemistry, often requiring non-biological catalysts or conditions, raises biocompatibility challenges. This limitation motivates the exploration of biomolecule-mediated adhesion, which leverages the specificity and innate compatibility of biological recognition motifs.

### 2.4 Surface biomolecule-mediated adhesion

Transitioning from synthetic chemical ligation, biomolecule-mediated adhesion represents a shift toward using evolutionarily refined recognition motifs. This strategy helps bridge synthetic constructs with native tissues by harnessing the intrinsic specificity, programmability, and biocompatibility of biological molecules. It primarily relies on two molecular scaffolds: proteins, which provide high-affinity binding and stimulus-responsive dynamics, and nucleic acids—particularly DNA—which offer exceptional sequence-dependent programmability for spatial control. Together, they empower the construction of prototissues with self-sorting architectures, integrated signal transduction, and adaptive behaviors that closely approximate the complexity of living systems.

#### 2.4.1 Protein-mediated specific adhesion

Protein-based molecular recognition systems utilize high-affinity, specific interactions to engineer programmable prototissue architectures. Their development illustrates a progression from generic high-affinity binding pairs toward dynamically regulated, biomimetic adhesion mechanisms.

The biotin-streptavidin interaction, characterized by its ultrahigh affinity (Kd ≈ 10^−15^ M) and tetravalent nature [66], serves as a foundational tool for robustly bridging protocells. The adhesion strength in such systems is concentration-dependent, enabling the design of tunable prototissues by optimizing streptavidin levels (e.g., 10–50 nM) to balance aggregation efficiency with membrane integrity [29]. However, the near-irreversible nature of this interaction limits its utility in applications requiring dynamic reorganization or adaptive responses, restricting its use largely to static scaffold construction.

Progressing toward greater biological fidelity, lectin-glycan systems emulate natural glycosphingolipid-mediated adhesion processes [93,94]. Multivalent lectins (e.g., LecA) can crosslink glycan-functionalized GUVs into tissue-like clusters [67]. A key advancement in this area involves engineering the membrane interface; for example, incorporating lipopolymers to mimic the glycocalyx introduces repulsive forces that counterbalance specific lectin–glycan binding, thereby improving the biomimetic fidelity of the interaction. Furthermore, integrating these interactions with responsive materials—such as thermoresponsive polysaccharidosomes that form reversible lectin-mediated networks [68] enables environmentally gated biosensing and other adaptive functions.

For supreme spatiotemporal precision, optogenetic protein pairs surpass chemically mediated systems. The iLID-Nano platform, for example, achieves rapid and reversible light-controlled adhesion through a blue light-induced conformational change that increases binding affinity by over 50-fold [72,95,96]. This capability has been leveraged to construct complex interactive systems, such as a "predator-prey" model where chemiluminescent sender GUVs activate iLID-based adhesion to Nano-coated receivers under blue light [69]. This system exhibits remarkable autonomy, self-regulating adhesion based on H_2_O_2_ concentration and undergoing disassembly upon signal depletion, while also facilitating bidirectional Ca^2+^ signaling upon contact (Fig. 2h) [71]. The sophistication further increases when orthogonal optogenetic modules (e.g., iLID–Nano with PhyB–PIF6) are integrated with DNA strand-displacement (DSD) circuits, enabling programmable assembly of multi-member communities and logic-gated signal processing [70] This convergence marks a significant leap toward prototissues with life-like communication and organizational intelligence.

#### 2.4.2 DNA-programmed assembly

Complementing protein-based strategies, DNA-programmed assembly exploits the predictable thermodynamics and sequence complementarity of DNA hybridization, offering advantages in spatial patterning, thermal reversibility, and nanoscale architectural control, thereby enabling the precision engineering of prototissue architectures [[73], [74], [75],^77^,^97^].

Early implementations faced significant hurdles. Liposome systems modified with DNA were prone to uncontrolled fusion or rupture due to membrane destabilization [98], while the synthesis of amphiphilic DNA block copolymers such as PMA-b-DNA often suffered from low coupling efficiency and product heterogeneity, limiting their functional versatility [99,100].

A pivotal innovation was the introduction of cholesterol-tagged DNA anchors, which allow passive insertion of DNA strands into pre-formed polymersome or liposome membranes, bypassing complex covalent chemistry and enabling asymmetric functionalization [73]. Thermal cycling of the system then drives fully reversible aggregation-dissociation cycles, effectively translating molecular recognition into macroscopic, reconfigurable network dynamics. Building on this, surface-functionalized polymer GUVs (pGUVs) achieve programmable assembly with precise control over cluster architecture via cholesterol-ssDNA patterning [75]. The inherent mechanical robustness of triblock copolymers in pGUVs prevents membrane fusion and supports stimuli-responsive networks capable of complex intra- and intercellular communication.

Beyond mediating adhesion, DNA nanotechnology has been repurposed to replicate structural and scaffolding functions within prototissues. Crosslinked DNA fibers can form biomimetic skeletal networks inside GUVs, enabling fine-tuning of prototissue stability and morphology to generate architectures such as honeycombs or multilayers [77]. Furthermore, DNA origami templates bring atomic-level precision to the spatial organization of liposomes. Zhao et al. demonstrated this by using sticky-ended square origami to direct liposomes into programmable 2D arrays, finite lattices, and chiral rings, with valence and flexibility encoded in the origami design [76]. Toehold-mediated strand displacement enables on-demand reversible oligomerization or disassembly of these structures, providing real-time control over tissue architecture.

Despite these advances, lipid- and polymer-based systems often lack the biomolecular crowding and compartmentalization properties of real cytoplasm. Coacervates, formed through LLPS, address this gap by creating highly crowded, biomimetic microenvironments [[101], [102], [103], [104], [105]]. The concept of all-DNA protocells has been demonstrated using coacervates functionalized with DNAzymes [74]. In these systems, catalytic substrate cleavage releases palindromic sequences that drive multivalent coacervate assembly through complementary interactions (Fig. 2i), creating a distinctive form of catalytically programmable prototissue formation.

In brief, protein-mediated adhesion excels in capturing dynamic biological responsiveness—from lectin–glycan interactions to optogenetic precision—but often requires complex protein engineering. DNA assembly complements this by offering deterministic spatial patterning through sequence programmability and straightforward thermal control. The convergence of these approaches, as seen in systems combining optogenetic proteins with DNA circuits [70], creates a powerful synergistic potential for engineering prototissues with life-like communication, reconfigurability, and organizational intelligence. These biomolecular strategies exhibit superior biocompatibility and functional sophistication compared to previous physicochemical methods. However, native tissues depend not only on cell–cell interactions but also on the ECM, which provides structural support, mechanical cues, and biochemical signaling. This critical dimension motivates the exploration of ECM-mimetic adhesion strategies.

### 2.5 ECM-mediated adhesion

In native tissues, higher-order functionality arises not only from cell–cell contacts but also from the dynamic interplay between cells and the surrounding ECM. The ECM provides a key architectural scaffold that organizes cells, transmits mechanical forces, and presents reservoirs of biochemical cues—principles evident in tissues such as cartilage and bone [[106], [107], [108], [109]]. Inspired by this biological paradigm, synthetic prototissue engineering has developed ECM-mimetic strategies to transcend the limitations of protocell-alone systems [47,78,[80], [81], [82],^110^]. Hydrogels have emerged as a central platform owing to their hydrous and porous microstructure, tunable mechanics, and capacity for bio-inspired functionalization, enabling partial recapitulation of key ECM attributes [111]. These approaches have progressed from providing simple spatial confinement to actively processing environmental inputs, thereby enhancing the modularity and responsiveness of prototissues.

Initial ECM-mimetic approaches primarily leveraged hydrogels as static, instructive scaffolds for spatial organization. A seminal demonstration involved the immobilization of enzyme-decorated coacervate vesicles within agarose hydrogels to form concentric tubular prototissues [80]. This architecture leveraged the hydrogel's porosity to establish controlled reaction-diffusion gradients, enabling logic-gated nitric oxide (NO) production that effectively inhibited platelet activation in vascular models (Fig. 2j). While this showcased the utility of hydrogels in imposing order and facilitating biochemical signaling, the static nature of the matrix limited dynamic adaptation and reconfiguration.

To overcome this rigidity, subsequent research has focused on engineering dynamic and responsive ECM analogs. A significant advancement was achieved using stimuli-responsive gelatin matrices to fabricate centimeter-scale prototissue materials (PTMs) [81]. These systems integrated multiple functions: the thermoresponsive matrix permitted elasticity modulation, while embedded photothermal nanoparticles enabled light-triggered heating. Together, these features produced emergent behaviors such as osmotic–thermal-gradient-driven morphogenesis and predator–prey-like interactions mediated by photothermal release of enzymatic substrates. Importantly, these PTMs supported bidirectional mechanochemical communication, enabling dynamic reconfiguration reminiscent of nutrient uptake and inter-tissue competition and thereby narrowing the functional gap with native tissues.

Extending beyond chemical and thermal signaling, the most advanced ECM-mimetic systems now replicate complex biomaterialization processes [82]. For instance, alkaline phosphatase-loaded colloidosomes embedded within alginate-methacrylate hydrogels have been engineered to direct spatially patterned calcium phosphate mineralization (Fig. 2k). Here, the hydrogel matrix functions not just as a scaffold but as an active regulator of ion gradients, guiding the spatiotemporal deposition of mineral. Remarkably, the system supports reversible calcification–decalcification cycles through modulation of matrix composition and colloidosome density. This establishes a synthetic ECM paradigm capable of directing long-range structural remodeling, echoing the roles of ECM in mineralized tissues.

In summary, ECM-mimetic strategies have evolved from passive spatial frameworks to interactive, stimulus-responsive platforms capable of processing multimodal environmental inputs. The progression from simple agarose confinement to dynamic gelatin matrices and, ultimately, to mineral-directing Alg-MA hydrogels reflects increasing functional sophistication. By integrating mechanical coordination with chemical communication, these strategies enable synthetic prototissues to exhibit emergent behaviors such as environmental morphogenesis and community-level interactions. Incorporating a synthetic ECM thus substantially narrows the functional gap between engineered prototissues and the complex adaptive behavior of living tissues.

Adhesion mechanisms form the primary regulatory layer through which prototissues acquire structural coherence, communication capacity, and coordinated collective behavior. Across the spectrum from physicochemical interactions to engineered molecular and biomolecular linkages, distinct parameters—including adhesion density, binding affinity, molecular specificity, and reversibility—determine how individual protocells pack, exchange signals, and reorganize in response to environmental cues. These determinants govern higher-order outcomes such as self-sorting of heterogeneous protocell populations, the establishment of protected reaction–diffusion microenvironments, and the emergence of coordinated functions ranging from synchronized contraction to morphogenetic shape change. By defining how units connect, stabilize, and interact, adhesion strategies create a foundational “connectivity architecture” that preconfigures the behavioral potential of prototissues. This connectivity dimension thus provides the essential basis upon which the second dimension—macroscopic spatial programming—can further shape, organize, and direct prototissue function.

## 3 The methods of controlling the geometry

While molecular adhesion defines connectivity, spatial programming governs collective geometry—the second dimension of the synergistic framework.

**Fig. 3:**
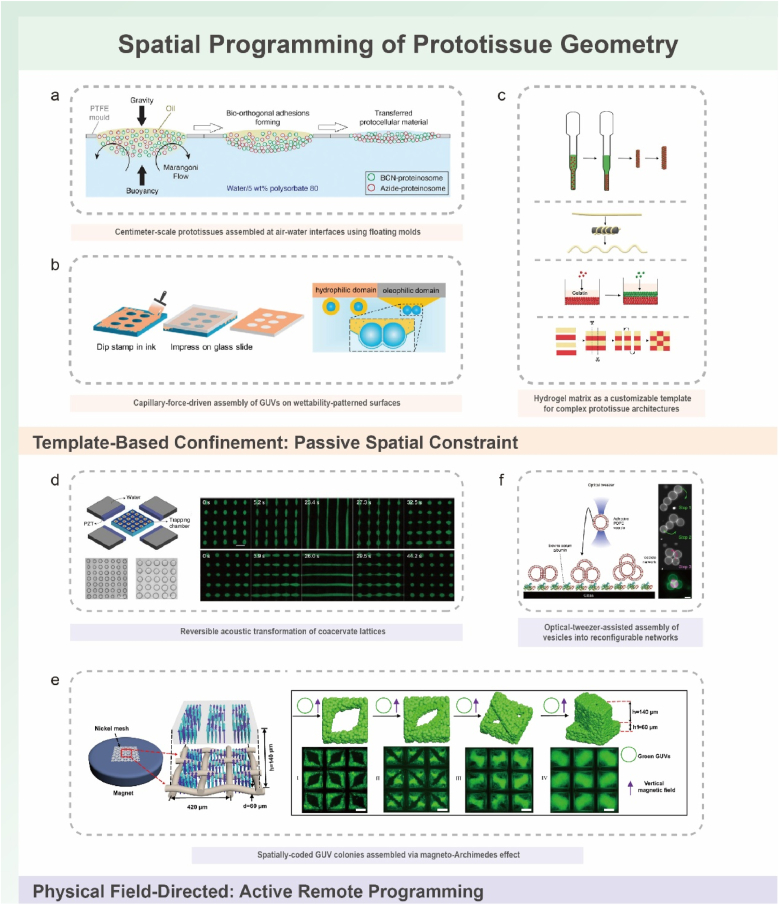
Spatial programming of prototissue geometry via template-based passive confinement and physical-field-directed active manipulation. (a–c) Template-based confinement: passive spatial constraint. (a) Floating mold assembly at the air–water interface, where proteinosomes are passively confined within PTFE molds and stabilized by bioorthogonal adhesion to form centimeter-scale prototissues. Reproduced with permission [62]. Copyright 2021, Wiley-VCH. (b) Wettability-patterned substrates, in which hydrophilic/oleophilic microdomains passively guide capillary-force-driven accumulation of GUVs into predefined surface patterns. Reproduced with permission [40]. Copyright 2023, American Chemical Society. (c) Hydrogel matrices as customizable templates, where patterned or layered gels provide static, ECM-like microenvironments that constrain protocell positioning and support the construction of complex 2D/3D architectures. Reproduced with permission [81].Copyright 2024, Wiley-VCH. (d–f) Physical field-directed organization: active remote programming. (d) Acoustic standing waves actively drive coacervate droplets into reversible 1D and 2D lattices, with field parameters controlling lattice spacing and pattern transformation. Reproduced with permission [53]. Copyright 2016, Springer Nature. (e) Magneto-Archimedes alignment, in which external magnetic fields and a nickel mesh create 3D magnetic potential wells that actively levitate and position diamagnetic GUVs into spatially coded colonies. Reproduced with permission [31]. Copyright 2022, Springer Nature. (f) Optical-tweezers-assisted assembly, where focused laser traps actively capture, move, and rearrange vesicles to build reconfigurable networks with single-protocell precision. Reproduced with permission [34]. Copyright 2018, Springer Nature. Overall, template-based methods impose static geometric constraints to define prototissue shape, whereas acoustic, magnetic, and optical fields actively and remotely program protocell positions in real time, enabling dynamic and reconfigurable spatial architectures.

Beyond inter-protocell adhesion at the molecular scale (Section 2), the establishment of controlled spatial organization determines how these interactions translate into higher-order collective behaviors. The spatial dimension links microscopic connectivity to emergent macroscopic architecture, shaping not only structural order but also mass transport, communication efficiency, and mechanical performance within prototissues. In synthetic systems, uncontrolled aggregation through manual mixing or centrifugation often produces amorphous assemblies that lack the hierarchical organization required for emergent biological functions [59,67,69,70,74,112,113]. To overcome this limitation, spatial-organization strategies for prototissues can be broadly categorized into template-guided, field-directed, and micromanipulation-based assembly approaches (Fig. 3). Each method offers distinct advantages in spatial precision, throughput, and functional adaptability (Table 2).

**Table 2:** Spatial programming strategies for constructing prototissues.

Spatial programming paradigm	Principle	Control factors	Advantages	Limitations
**Template-guided assembly**	Physical confinement using floating molds, wettability patterns [40,62,64], hydrogel matrices defining geometric boundaries or diffusion landscapes [35,[46], [47], [48],[78], [79], [80], [81], [82]]	Template geometry, interfacial tension, mold material, hydrogel composition, hydrogel porosity, crosslinking density	Centimeter-scale architectures, long-term stability, hierarchical organization via diffusion guidance, compatibility with reaction–diffusion patterning	Spatial-resolution limits imposed by template fidelity, difficulty in reconfiguration once fixed, slow mass transfer in hydrogels, constraints on dynamic assembly
**Field-directed assembly**	Acoustic radiation pressure–based patterning [30,32,53,[114], [115], [116]], diamagnetic levitation [31,33,[116], [117], [118]], optical momentum trapping [34]	Acoustic frequency and amplitude, medium density, magnetic susceptibility, field gradient, laser power, refractive index contrast	Non-contact reversible manipulation, millisecond response, formation of dynamic lattices and tunable architectures, activation or modulation of local reactions	Specialized equipment requirements, low throughput of optical-tweezers manipulation, limited spatial resolution of acoustic and magnetic fields
**Micromanipulation-based assembly**	Micropipette handling of protocells [36,45,47,49,51,84],microfluidic confinement [35,63,79], droplet interface bilayer (DIB)-based 3D printing [37,[42], [43], [44],^46^,^50^,^119^]	Pipette diameter and pressure, microchannel geometry, flow rate, printing path, droplet composition	Highest spatial precision, deterministic control of compartment number and distribution, suitability for heterogeneous and hierarchical assemblies, construction of centimeter-scale 3D printed tissues	Low throughput of micropipettes, microfluidic constraints imposed by channel design, printing speed limits for single-nozzle deposition, limited free-form reconfiguration after fabrication

### 3.1 Template method

Template-guided assembly provides direct geometric control over prototissues by confining protocells within predefined architectures. This strategy operates through two principal paradigms: interface-confined organization, such as floating molds, which enables macroscopic structuring; and ECM-mimetic hydrogel scaffolds, which introduce microenvironmental coordination and enhanced biological fidelity. Each approach offers distinct advantages in scalability, spatial resolution, and compatibility with downstream functionalization.

#### 3.1.1 Floating mold technique

The floating mold technique confines buoyant protocells at fluid interfaces within precisely fabricated templates, enabling the construction of well-defined macroscopic architectures. A notable advancement employed laser-cut poly(tetrafluoroethylene) molds to organize bioorthogonally functionalized proteinosomes into centimeter-scale protocellular assemblies at the air–water interface [62,64].These constructs exhibited tailored geometries (Fig. 3a) [62], exceptional aqueous stability exceeding six months, and sophisticated hierarchical organization through surfactant-mediated oil removal. Importantly, defined geometries allowed predictable reaction–diffusion gradients and environment-responsive signal transduction —functionalities that are unattainable in amorphous, randomly aggregated systems. Consequently, such templates provide a valuable platform for studying nonequilibrium biochemical sensing, as the controlled architecture enables precise spatial mapping of gradient-dependent responses.

Complementing these rigid templates, capillary-driven assembly employs wettability-patterned substrates to guide protocell organization through interfacial forces. On glass coverslips patterned with hydrophilic polyvinyl alcohol domains, vesicle aggregation is confined to specific regions, while oleophilic areas generate capillary forces that promote dense two-dimensional packing (Fig. 3b) [40]. Although this stamp-based approach enables rapid prototyping of surface patterns, it remains inherently limited to planar configurations due to its dependence on interfacial constraints. Both floating mold and capillary techniques demonstrate how physical confinement alone can orchestrate prototissue formation, yet they offer limited dynamic interaction with the encapsulated protocells compared to hydrogel-based approaches.

#### 3.1.2 Gel-embedded scaffolds

In contrast to the static confinement provided by floating molds, gel-embedded scaffolds use ECM-mimetic hydrogels to create programmable microenvironments that actively contribute to prototissue function [78,80,81]. Early demonstrations used agarose/alginate matrices to entrap coacervate microreactors, where hydrogel porosity established diffusion barriers that enabled spatially organized photocatalytic–peroxidation cascades with directional fluorescence propagation [78]. This modular design facilitated physical fusion of functional units, establishing foundational principles for scalable prototissue architectures.

Subsequent advances leveraged the compositional tunability of hydrogels to achieve more sophisticated functions. Concentric agarose layering enabled vascular-mimetic hierarchies in which spatial segregation of enzyme-decorated protocells produced glucose- or hydroxyurea-triggered NO [80]. The resulting radial diffusion profiles effectively inhibited platelet activation in blood-filled lumens, demonstrating therapeutic potential in anticoagulation. Further developments introduced stimuli-responsive matrices, such as gelatin with sacrificial CaCO_3_ templates, allowing on-demand customization of prototissue morphology and modular assembly (Fig. 3c) [81]. These systems displayed emergent behaviors—including bidirectional mechanochemical communication, osmotic–thermal morphogenesis, and predator–prey interactions via photothermal substrate release—highlighting how matrix-mediated feedback enables multicellular-like adaptability.

Overall, the progression of template strategies reflects a shift from passive geometric confinement to active microenvironmental regulation. While interface-based techniques excel in centimeter-scale structuring with defined geometry, gel-embedded systems uniquely recapitulate tissue-level functionalities through integrated spatiotemporal coordination. Future integration with 4D bioprinting and enzyme-programmable matrices is expected to yield reconfigurable prototissues capable of dynamic adaptation to physiological cues, further narrowing the gap between synthetic constructs and living tissues [120].

### 3.2 Physical field-directed organization

Template methods produce predefined structures dictated by scaffold geometry, whereas physical fields enable guided self-organization within tunable energy landscapes. This shift allows real-time reconfiguration and scalable growth of prototissues, where additional protocells can be integrated without redesigning the underlying template. By harnessing intrinsic protocell properties—such as acoustic contrast, diamagnetic susceptibility, and refractive index mismatch—acoustic, magnetic, and optical fields provide remote, non-invasive spatial manipulation, overcoming the static constraints of template-based approaches.

#### 3.2.1 Acoustic field patterning

Acoustic field technology leverages density and compressibility contrasts between protocells and their medium to generate non-contact radiation forces through standing wave pressure fields [114,121,122]. This approach offers sub-millisecond positional control with micron-scale precision, providing substantial potential for high-throughput prototissue engineering.

Building on these capabilities, acoustic fields have been used to construct complex protocell arrays and prototissues [30,53,115]. Seminal work demonstrated the assembly of PDDA/ATP coacervates into defect-free 2D arrays via standing waves (Fig. 3d) [53]. Through precise frequency modulation, researchers achieved *in situ* droplet coalescence, programmable enzyme loading, and reversible shape transformations, establishing acoustic fields as a powerful method for generating adaptive microreactor networks. However, these early systems were constrained to coacervates with intrinsically high acoustic contrast.

A pivotal innovation addressed this constraint through engineering sucrose/glucose density gradients to overcome the low intrinsic contrast of GUVs. This enabled precise fabrication of linear, square, and triangular GUV arrays with controlled lattice spacing, facilitating the formation of hemifused colonies with enhanced functional capabilities [30]. The principal strength of acoustic manipulation lies in its dynamic programmability: unlike static templates, acoustic fields permit real-time reconfiguration of array patterns through field modulation. Functionally, these reconfigurable assemblies have supported enzyme-mediated signaling cascades and hybrid protocell–living cell consortia, including melittin-triggered cancer cell killing and bacterial gene induction.

While most arrays generated in solution yield 1D or 2D architectures, the incorporation of hydrogel droplets enables construction of more complex 3D structures. Recent innovations have integrated bulk acoustic wave (BAW) technology with microfluidics and hydrogel matrices to assemble sophisticated 3D constructs [116]. A representative system organizes cell-laden hydrogel droplets—produced by microfluidics—into defined 3D patterns under tunable acoustic fields. This approach formed MCF-7 spheroids or linear chains at rates up to 6000 units per minute while preserving high viability and accelerating tissue maturation. Although demonstrated using mammalian tumor cells, the methodological framework—combining acoustic precision with microfluidic encapsulation and hydrogel scaffolding—provides a directly transferable strategy for high-throughput 3D prototissue fabrication. This integrated approach marks a transition from simple 2D droplet arrays to dimensionally stable, functionally complex tissue constructs with enhanced biomimetic potential.

#### 3.2.2 Magnetic field alignment

Compared to acoustic fields, magnetic manipulation offers a complementary non-invasive approach that is particularly advantageous for constructing complex 3D tissue-like architectures. Traditional strategies based on magnetic particle conjugation encounter limitations such as cytotoxicity and interference with downstream biological assays [123]. The emergence of the magneto-Archimedes (Mag-Arch) effect has shifted this paradigm by exploiting paramagnetic media to manipulate diamagnetic objects via negative magnetophoresis, enabling contactless assembly under low-field conditions without labeling protocells [124].

The implementation of the Mag-Arch effect using metallic meshes has enabled the assembly of prototissue structures with tailored 3D architectures and the investigation of how spatial organization dictates collective behavior. Initial studies used stainless-steel meshes to assemble 3D GUV colonies with defined lattice spacing and, through modulation of magnetic field distributions, generated spatially encoded structures such as layered and grid-like arrangements [33]. This approach was further scaled by Zhang et al., who employed nickel mesh templates to fabricate thousands of multicomponent prototissues capable of executing enzymatic cascades that induced vasodilation in living tissue (Fig. 3e) [31].

Beyond structural fabrication, magnetic spatial programming has proved valuable for probing ecological principles in synthetic communities. Li et al. constructed spatially coded three-species consortia and demonstrated that particular arrangements (e.g., A'CB) disrupted the feedback required for pH oscillations, thereby altering community-level dynamics [117]. This work highlights magnetic spatial coding as not only an assembly tool but also a mechanistic approach for elucidating how spatial positioning modulates communication and metabolic interaction within prototissues.

While metallic meshes offer high precision, their rigid architecture may damage constructs during detachment and limit biomedical applicability. Recent advances in tissue engineering have adopted scaffold-free magnetic array systems that pattern living cells by modulating paramagnetic reagent concentrations and magnetic field configurations without physical contact [118]. Although these studies were performed with natural cells, the methodological framework is directly transferable to future prototissue engineering, offering greater flexibility and reducing mechanical constraints.

#### 3.2.3 Optical tweezers-based positioning

While acoustic and magnetic methods enable versatile protocell manipulation, optical tweezers offer unmatched submicrometer resolution and dynamic control, achieving precise 3D positioning through refractive-index–driven photon momentum transfer. This non-contact method supports the assembly of reconfigurable vesicle networks with exceptional precision, exemplified by user-defined architectures such as trigonal lattices and multilayered pyramids interconnected by VIMs (Fig. 3f) [34]. The fine control provided by optical tweezers permits reversible adhesion modulated by environmental parameters such as NaCl concentration, while incorporation of photothermal nanoparticles enables light-triggered cargo exchange and protein expression. Despite these advantages, several constraints limit broader implementation. Manual vesicle manipulation restricts scalability to small assemblies, and network reconfiguration remains largely unidirectional. In addition, the need for sucrose encapsulation to establish refractive-index contrast introduces osmotic constraints that may impair physiological relevance. Future improvements may include integrating bioorthogonal adhesion systems such as biotin–streptavidin or DNA-based recognition to reduce salt dependency, while engineered transmembrane proteins could enhance inter-vesicle communication.

Collectively, physical field technologies address spatial-organization challenges through complementary strengths: acoustic fields provide high-throughput patterning at millimeter scales; magnetic manipulation enables formation of complex 3D prototissue architectures; and optical tweezers deliver submicrometer precision for dynamic reconfiguration. This technological triad forms a versatile toolkit for constructing hierarchical synthetic protissues, though limitations remain in scaling optical approaches, reducing magnetic reagent interference, and improving acoustic resolution. Future progress will likely arise from multimodal integration—such as acoustic pre-patterning combined with optical refinement or magnetic positioning coupled with photoresponsive adhesion—potentially enhanced by machine-learning–based field optimization. Such synergistic strategies may ultimately narrow the gap between synthetic prototissues and the functional complexity of native tissues.

### 3.3 Micromanipulation-based fabrication

While field-directed strategies provide non-contact, high-throughput patterning of protocell arrays, they often lack single-unit precision. To address this gap, micromanipulation-based fabrication employs direct physical intervention to achieve fine control over assembly, linking molecular-scale design with macroscopic tissue engineering. This paradigm encompasses three complementary techniques: micropipette-assisted assembly for bespoke hierarchical structures, microfluidic confinement for high-throughput generation of calibrated units, and 3D bioprinting for programmable construction of centimeter-scale, geometrically defined architectures. Together, these methods offer a versatile toolkit to reconcile the “precision–throughput–scale” paradox in prototissue engineering.

#### 3.3.1 Micropipette-assisted assembly

**Fig. 4:**
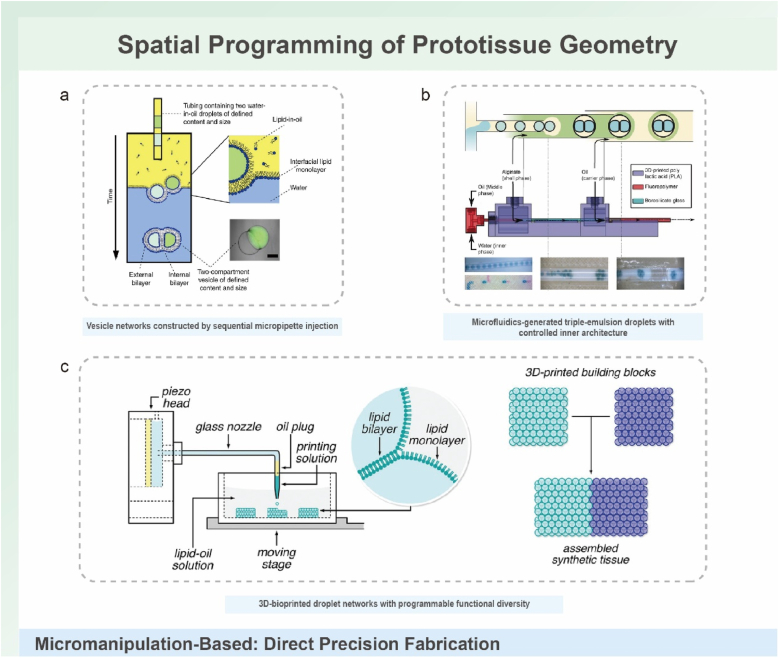
Micromanipulation-based strategies for direct precision fabrication of prototissue architectures. (a) Sequential micropipette injection used to construct multi-compartment vesicles with controlled sizes and defined internal compositions, enabling branched or networked protocell geometries. Reproduced with permission [51]. Copyright 2014, Springer Nature. (b) Microfluidic production of triple-emulsion droplets, where nested water–oil–water emulsions are generated in a multilayer device to yield protocells with tunable internal structure and high fabrication throughput. Reproduced with permission [35]. Copyright 2016, Wiley-VCH. (c) 3D droplet bioprinting of protocell networks, in which picoliter droplets are dispensed into an oil phase and brought into contact to form DIBs generated by forced lipid monolayer contact, assembling modular droplet clusters into synthetic tissues with programmable connectivity. Reproduced with permission [46]. Copyright 2022, Wiley-VCH. Overall, micromanipulation techniques enable deterministic, voxel-level placement of protocell units, providing unmatched spatial precision for building complex prototissue architectures.

Micropipette techniques represent a foundational approach that balances operational simplicity, minimal instrumentation, and high precision in constructing hierarchically organized artificial cell architectures [45,47,49,51]. The canonical method involves sequential injection of aqueous droplets into an oil phase, where density-driven encapsulation forms multi-compartment vesicles (Fig. 4a) [51].TThis enables precise co-localization of biochemical components—for example, GOx- and HRP-loaded droplets—while DIB formation segregates reaction pathways, and incorporation of transmembrane pores such as α-HL permits controlled inter-compartment communication.

However, the manual nature of this approach limits scalability and architectural complexity, typically restricting assemblies to ∼5 compartments due to pipetting constraints. Although DIBs are theoretically reconfigurable, manual assembly renders network modification laborious and impractical. To overcome this barrier, recent advances integrate hydrogel matrices. Injecting nanoliter liposome droplets into agarose-encapsulated oil compartments generates stable hierarchical architectures spanning proto-organelles, protocells, and interconnected prototissues [47]. This hydrogel-assisted micropipetting combines the positional fidelity of manual manipulation with the stability and scalability of a biomimetic scaffold, thereby addressing a central limitation of conventional droplet techniques.

#### 3.3.2 Microfluidic confinement

Surpassing the throughput and automation limitations of manual pipetting, microfluidic technology enables precise, high-throughput spatial organization of artificial cells through automated flow-focusing geometries [35,39,63,73,79]. This platform excels at generating monodisperse, emulsion-based constructs with fine control over internal architecture. A representative example is the triple-emulsion system, which produces hydrogel-encapsulated constructs containing a defined number of internal aqueous droplets (1–10 per unit) (Fig. 4b) [35]. These assemblies preserve DIB-mediated communication while benefiting from a robust hydrogel shell that provides mechanical stability (>50 kPa) and maintains diffusive exchange, overcoming the intrinsic fragility of unsupported droplet networks.

Further expanding functional complexity, water–oil–water (W/O/W) emulsion platforms generate bioorthogonally functionalized protocells within bioinert membranes [63]. Poly(dimethylsiloxane) devices containing multiple flow-focusing junctions offer precise control over stoichiometry and spatial arrangement of different protocell populations. By tailoring channel geometry and flow parameters, researchers can replicate tissue-level structural asymmetries such as Janus configurations. The ability to produce complex, heterogeneous building blocks in a high-throughput manner establishes microfluidics as an indispensable platform for regenerative medicine and modular prototissue engineering.

#### 3.3.3 3D bioprinting

3D bioprinting overcomes the scale constraints of manual micropipetting and the geometric limitations of microfluidic channels, providing software-directed spatial control at millimeter-to-centimeter scales. It exploits DIBs as “tissue solder,” formed when lipid monolayers surrounding aqueous droplets fuse upon contact in an oil/lipid medium [37,[41], [42], [43], [44],^46^,^50^,^119^].

Early demonstrations established the feasibility of printing self-supporting tissues comprising thousands of picoliter droplets that assembled into hexagonal lattices via DIBs, exhibiting osmolarity-driven folding reminiscent of plant movements. However, large networks (>100 droplets) often displayed structural defects and irregular conductive pathways that compromised functional resolution [50]. A critical advance came with the identification of the equilibrium DIB contact angle (θDIB) as a key parameter governing packing geometry [37]. When tuned to the theoretical threshold of 35.3°, droplets formed regular hexagonal lattices with minimal imperfections, enabling single-droplet-wide conductive pathways and substantially improved functional resolution.

To address inherent speed limitations of sequential droplet printing, modular fabrication strategies have been introduced. In this approach, functional subunits are printed under optimized—sometimes mutually incompatible—conditions (e.g., temperature), then integrated via interfacial bilayers into centimeter-scale tissues containing >10,000 compartments (Fig. 4c) [46]. This method accelerates throughput and broadens functional diversity, enabling incorporation of Boolean logic gates, magnetic responsiveness, and encapsulated living cells. These advances position 3D bioprinting as a transformative platform for constructing large-scale prototissues with programmable geometry and biologically relevant functionality.

In summary, the three micromanipulation strategies provide complementary capabilities. Micropipette-assisted assembly is well suited for prototyping and probing communication mechanisms in small, customized architectures (typically a few to dozens of compartments). Microfluidic confinement enables high-throughput production of standardized, complex building blocks—such as Janus particles and multicore emulsions—suitable for statistically robust studies and modular tissue construction. Finally, 3D bioprinting uniquely supports bottom-up fabrication of large-scale, macroscopically functional tissues with programmable geometry, suited for modeling organ-level behaviors such as coordinated actuation and long-range signal propagation. Strategic selection and integration of these methods remain central for advancing prototissues toward greater architectural and functional sophistication.

The spatial-programming strategies outlined in Sections 3.1–3.3 collectively form a versatile toolkit, each with distinct strengths. Template-guided assembly enables centimeter-scale, custom-shaped architectures but is limited by predefined geometry. Field-directed assembly offers dynamic, non-contact manipulation, with acoustic fields suited for high-throughput 2D patterning, magnetic fields for complex 3D architectures, and optical tweezers for submicrometer precision. Micromanipulation methods add architectural customization and scale, from bespoke micropipetting to modular microfluidics and large-scale 3D bioprinting. Together, these strategies enable increasingly hierarchical and functional prototissues.

### 3.4 A dual-dimensional synergistic framework for rational prototissue design

The preceding sections summarize a broad toolkit for prototissue construction, ranging from molecular-scale adhesion mechanisms to macroscale spatial programming. To advance from empirical assembly to rational engineering, these strategies must be integrated within a coherent framework. In this section, a Dual-Dimensional Synergistic Framework is presented in which prototissue architecture and function emerge from the combined influence of two design dimensions. Inter-protocell adhesion determines the form and behavior of interfacial contacts, and spatial programming organizes these interactions into macroscopic structures. Together, these dimensions offer both a conceptual lens for understanding current systems and a practical guide for designing prototissues with targeted properties.

Inter-protocell adhesion forms the molecular basis for structural stability, communication, and responsiveness. Adhesion strategies range from strong and persistent linkages, including click chemistry and biotin–streptavidin binding, to reversible systems that allow structural reconfiguration, such as DNA hybridization, optogenetic pairs, and dynamic covalent bonds. Differences in permeability at these interfaces influence the fidelity of signal exchange. Non-specific contacts, such as membrane fusion or electrostatic interactions, allow broad molecular transfer, whereas engineered channels, including α-HL pores or DNA-based recognition, enable more selective signaling. Adhesion mechanisms also differ in their compatibility with biological environments. Protein–ligand interactions, lectin–glycan recognition, and ECM-inspired matrices align well with biomedical applications, while abiotic linkages, including DIBs and synthetic covalent bonds, provide stability suited to *in vitro* systems.

Spatial programming acts as the architectural system that translates molecular interactions into collective behaviors. The arrangement of protocells governs how signals propagate through a prototissue. Random assemblies often restrict molecular transport to local diffusion, whereas programmed geometries, such as acoustic lattices or 3D-printed networks, create predictable pathways that support multi-step reactions and coordinated responses. Spatial arrangement also affects mechanical behavior. Isotropic assemblies respond uniformly to external stimuli, while anisotropic or layered structures can generate bending, twisting, or other direction-dependent deformations. In addition, specific spatial programming methods align with different application goals. Microfluidic and printing techniques support microphysiological model construction, template-based methods suit implantable constructs, and field-directed assembly can be applied in engineered metamaterials.

The relationship between the two dimensions may be independent or cooperative. Some systems rely mainly on spatial patterning without specialized adhesion, as seen in acoustically assembled transient arrays, while others use molecular recognition to achieve self-sorting without external positioning. The most advanced behaviors arise when both dimensions are integrated. For example, acoustic alignment combined with calcium-mediated hemifusion allows GUV colonies to retain ordered structures and perform coordinated, muscle-like contractions [32]. Likewise, combining 3D bioprinting with reversible DNA-mediated adhesion creates large constructs that can reconfigure internal connectivity while maintaining overall form.

In summary, the Dual-Dimensional Synergistic Framework provides a structured foundation for the rational design of prototissues. Adhesion determines how protocells connect and exchange information, and spatial programming shapes these connections into functional tissue-like architectures. Understanding when the two dimensions should be applied independently and when they should be integrated will be essential for constructing prototissues with increasing levels of adaptiveness and complexity. This perspective supports the development of next-generation systems for use in biomedicine, soft robotics, and intelligent materials.

## 4 Stimuli-responsive behaviors in prototissues

Adhesion mechanisms and spatial programming establish the structural basis of prototissues, but their functional sophistication emerges through stimulus-responsive behaviors. Physical inputs—including osmotic pressure, temperature, light, and magnetic fields—drive adaptive reconfiguration, while mechanochemical coupling introduces bidirectional transduction between mechanical state and chemical function (as summarized in Table 3). These capabilities collectively enable synthetic assemblies to approximate the dynamic responsiveness of living tissues.

**Table 3:** Stimuli-responsive behaviors and mechanochemical coupling in prototissues.

Category	Stimuli/systems	Mechanical response modes	Underlying mechanisms	Functional/application relevance
**Physical stimuli → mechanical behaviors**	Osmotic gradients [31,33,50,81], thermal cues [48,60,81], light-induced photothermal stimulation [48,64,81], magnetic-field modulation [46,48,57,58]	Reversible swelling/deswelling, expansion–contraction, bending or folding, directed movement	Osmotically driven water flux, thermoresponsive matrix transitions, photothermal softening or shrinkage; magnetic-nanoparticle-driven force transduction	Programmable deformation, controllable locomotion, soft-robotic actuation
**Chemical → mechanical (chemically driven deformation)**	ATP-fueled actin polymerization [32],calcification reactions (e.g., CaP deposition) [82], pH-driven hydrogel assembly inside proteinosomes [60]	Contraction, curvature formation, anisotropic bending, modulation of thermal contraction in pH-sensitive gel networks	ATP-driven actin polymerization generating contractile forces, CaP deposition altering stiffness, pH-sensitive chemical reactions regulating hydrogel network assembly and mechanical responsiveness	Cytoskeleton-like contractility, morphogenesis-inspired shape programming, chemical regulation of thermal responsiveness
**Mechanical → chemical (mechanically regulated chemistry)**	Photothermal bursting of substrate-loaded protocells [81],photothermal contraction reducing membrane permeability [60,64]	Rupture and release, shrinkage, permeability decrease	Photothermal heating–induced membrane rupture, contraction-driven reduction of internal porosity and diffusive flux	ON-switch activation of enzymatic cascades, OFF-switch gating of reactions, spatiotemporal control of chemical pulses

### 4.1 Physically driven mechanical adaptation

Prototissues transduce environmental cues into coordinated mechanical outputs by integrating stimuli-responsive materials. This adaptive functionality, emerging from the collective interplay of constituent protocells, enables prototissues to mimic lifelike behaviors such as programmed deformation and directed locomotion.

#### 4.1.1 Osmotically programmed deformation

Osmotic gradients provide a fundamental driving force for prototissue reconfiguration, leveraging water flux to generate collective mechanical work. While individual GUVs are prone to rupture under osmotic stress [125], densely packed colonies exhibit emergent stability. This stability arises from collective mechanical reinforcement, where hypertonic compression generates elastic restorative forces, preventing catastrophic failure [33].

**Fig. 5:**
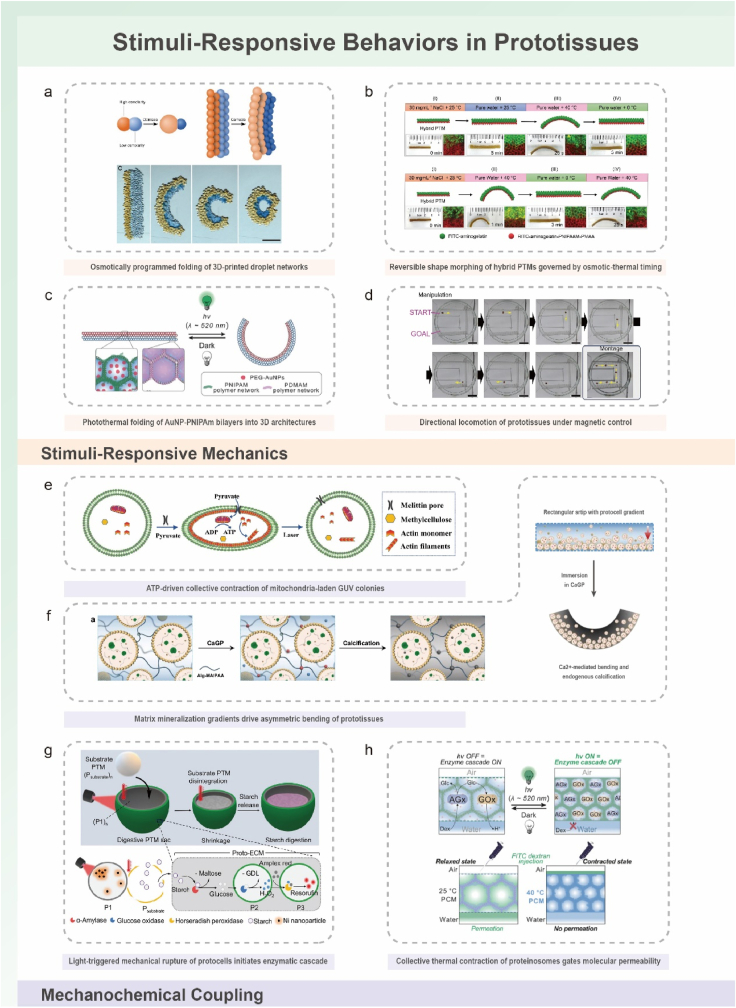
Stimuli-responsive behaviors in prototissues, encompassing externally triggered mechanical responses (a–d) and mechanochemical coupling (e–h). (a–d) Stimuli-responsive mechanical behaviors. (a) Osmotically programmed folding of 3D-printed droplet networks, where solute gradients induce coordinated curvature and shape transitions. Reproduced with permission [50]. Copyright 2013, American Association for the Advancement of Science. (b) Reversible morphing of hybrid PTMs governed by osmotic–thermal timing, enabling sequential bending, flattening, and reconfiguration. Reproduced with permission [81].Copyright 2024, Wiley-VCH. (c) Photothermal folding of AuNP–PNIPAm bilayers, in which light-induced polymer collapse drives asymmetric contraction and 3D architectural transformation. Reproduced with permission [64]. Copyright 2025, Wiley-VCH. (d) Magnetic-field-guided locomotion, where embedded magnetic components enable controlled translation and directional movement of prototissues under rotating or gradient fields. Reproduced with permission [57]. Copyright 2024, Wiley-VCH. (e–h) Mechanochemical coupling: bidirectional transduction between mechanical deformation and chemical activity. (e) Metabolism-driven collective contraction, where optically activated mitochondria produce ATP that triggers cytoskeleton-like contraction in GUV colonies. Reproduced with permission [32]. Copyright 2022, Wiley-VCH. (f) Mineralization-induced bending, in which Ca^2+^-mediated calcification gradients impose asymmetric stiffness, producing sustained curvature in prototissue strips. Reproduced with permission [82]. Copyright 2025, Springer Nature. (g) Mechanical-to-chemical transduction via photothermal rupture, where photothermal heating causes protocells containing starch substrate to mechanically burst, releasing cargo that initiates a multi-step enzymatic cascade. Reproduced with permission [81]. Copyright 2024, Wiley-VCH. (h) Chemical-to-mechanical regulation of reaction pathways, where photothermal-induced proteinosome contraction reduces membrane permeability, thereby suppressing or shutting down an enzyme cascade by limiting reagent transport. Reproduced with permission [64]. Copyright 2025, Wiley-VCH.

Beyond stability, osmotic forces can be harnessed for programmable deformation [31,50,81]. Engineered spatial organization, such as in magnetically assembled tricomponent prototissues, directs osmotic energy into density-dependent expansion–contraction cycles, enabling targeted deformation of distinct domains [31] Advancing further, 3D-printed networks of osmotically distinct droplets transform water exchange into complex, spontaneous folding motions (Fig. 5a) [50]. A key innovation to overcome the irreversibility inherent in such systems is the incorporation of hydrogel matrices. Gelatin-based prototissues achieve fully reversible, cyclable contraction and expansion, mirroring dynamic hydration responses in physiological tissues and enabling the study of multicellular osmotic signaling (Fig. 5b) [81].

#### 4.1.2 Thermally activated shape morphing

Thermoresponsive polymers, particularly poly(N-isopropylacrylamide) (PNIPAm), serve as primary actuators for thermal shape-morphing in prototissues. The volume phase transition of PNIPAm at its Lower Critical Solution Temperature (LCST) provides an effective mechanism to convert thermal energy into macroscopic work.

Collective behavior is demonstrated in proteinosome spheroids, where bio-orthogonal adhesion ensures synchronized volume changes across assemblies (>170 units), achieving ∼45 % contraction [60]. Patterning material composition shifts these responses from uniform contraction to programmable kinematics. Engineering differential crosslinking densities or combining polymers with distinct shrinkage coefficients produces reversible bilayer curling and related 3D shape changes [48]. Furthermore, the interplay between thermal and osmotic stimuli indicates that deformation kinetics depend not only on material composition but also on stimulus timing. Simultaneous triggering produces synergistic bending, highlighting opportunities for finer mechanical control based on stimulus sequencing (Fig. 5b) [81].

#### 4.1.3 Optical and magnetic stimuli for programmable prototissue functionality

Light and magnetic fields offer spatiotemporal precision that exceeds the global nature of osmotic and thermal cues, enabling targeted actuation and guided navigation.

Photothermal systems incorporating plasmonic nanoparticles convert light into localized heat, inducing asymmetric shrinkage to execute predefined folding or bending in selected regions (Fig. 5c) [48,64]. Magnetic manipulation provides a complementary, non-contact modality. Embedded nanoparticles or ferromagnetic elements enable prototissues to be propelled, transport cargo, or function as untethered grippers, often in combination with temperature-responsive materials for multimodal actuation (Fig. 5d) [42,48,57].

Physical stimuli thus provide prototissues with a diverse repertoire of adaptive behaviors. Osmotic and thermal inputs primarily drive volumetric changes and programmable folding—relevant to soft actuators and environmental sensors—whereas optical and magnetic cues enable precise spatial control crucial for targeted delivery and miniaturized robotics.

However, these responses are largely determined by the passive properties of constituent materials, limiting capacities for signal integration, memory, and autonomous decision-making. This limitation motivates the shift toward mechanochemical coupling, where mechanical state and biochemical activity influence each other to support higher-order functionality.

### 4.2 Mechanochemical coupling in prototissues

Physically driven adaptation describes how external stimuli induce structural deformation and collective motion in prototissues. In contrast, mechanochemical coupling establishes bidirectional transduction between mechanical and chemical domains, whereby mechanical forces and chemical reactions mutually influence one another. This coupling represents a higher level of functional integration, linking the structural adaptability of spatial organization to the biochemical responsiveness of molecular interactions. Through mechanochemical transduction, prototissues can convert local mechanical stress into biochemical activity or, conversely, generate motion and force from internal chemical energy—mirroring behaviors observed in living tissues.

#### 4.2.1 Chemical-to-mechanical transduction

This pathway transforms intrinsic chemical energy into macroscopic work, evolving from externally triggered systems to those exhibiting metabolic autonomy.

The foundational concept is exemplified by systems in which enzymatic reactions modulate the properties of stimuli-responsive matrices. For instance, the *in situ* formation of a peptide hydrogel within proteinosomes, triggered by an enzymatic cascade, fine-tunes thermally driven volume contractions, establishing a direct link between internal chemical signals and mechanical output [60]. A major advance is the integration of active metabolism to achieve energy autonomy. Mitochondria-loaded GUVs couple ATP production from pyruvate metabolism to fuel actin network polymerization (Fig. 5e) [32], directly transducing metabolic energy into sustained, reversible shape changes. Further sophistication arises from using chemical gradients to encode mechanical asymmetry. Inspired by biomineralization, spatially controlled enzymatic deposition of calcium phosphate in hydrogels generates intrinsic stiffness gradients (Fig. 5f) [82], driving predictable asymmetric bending and demonstrating how reaction–diffusion processes can imprint mechanical behavior into material structure.

#### 4.2.2 Mechanical-to-chemical regulation

The inverse paradigm treats the physical state of the prototissue as a dynamic regulator of its biochemical activity, enabling spatially and temporally precise control over reaction pathways.

A core mechanism involves using collective deformation to modulate molecular transport. Thermoresponsive contraction of proteinosome membranes reduces effective pore size, restricting substrate diffusion and thus downregulating encapsulated enzymatic activity in a reversible manner [60]. This establishes a simple yet powerful feedback loop between structure and function.

Photothermal nanomaterials further localize this control. By converting light into confined heat, they induce mechanical changes—such as membrane rupture or polymer mesh collapse—in specific subpopulations (Fig. 5g and h) [64,81], enabling targeted release or selective gating of enzymatic activity with high spatial precision.

Mechanochemical coupling in prototissues, though still emerging, shows a clear progression from externally modulated systems to constructs capable of increasing autonomy. The use of stimuli-responsive hydrogels (e.g., PNIPAm, gelatin, Alg-MA) and metabolic organelles provides a direct link between chemical states and mechanical properties. Current studies demonstrate that chemical energy—from enzymatic reactions, metabolism, or mineralization—can drive contraction, bending, and actuation; conversely, mechanical deformation can gate biochemical activity by modulating permeability and diffusion. These capabilities suggest opportunities for self-regulating drug delivery systems that respond to metabolic cues or soft robotic constructs capable of energy-autonomous, chemically guided locomotion.

However, present mechanochemical behaviors remain limited, often constrained to isotropic swelling/deswelling. The dependence on hydrogel-based matrices underscores the need for broader material strategies. Future advances will require engineering more complex mechanical outputs (e.g., twisting, peristalsis) and integrating synthetic biological circuits to achieve multi-input, logic-gated feedback control. Such developments will be essential for creating prototissues that not only respond to environmental cues but adapt and refine their behavior over time, further narrowing the gap between synthetic assemblies and living tissues.

## 5 Signal communication in prototissues

Beyond mechanical adaptability (Section 4), coordinated prototissue function requires mechanisms for efficient biochemical communication. Such communication relies on two complementary modes: passive diffusion across membranes and active, addressable signaling through engineered pores and channels. The choice between these mechanisms governs the spatiotemporal precision, specificity, and computational sophistication of collective responses, enabling behaviors from metabolic coupling to neuromorphic processing.

### 5.1 Direct transmembrane diffusion

Direct transmembrane diffusion serves as a ubiquitous and foundational communication mode, driven by concentration gradients without specialized molecular machinery. Its efficiency and selectivity, however, depend on the membrane properties of constituent protocells, leading to an evolutionary trajectory in synthetic design.

**Fig. 6:**
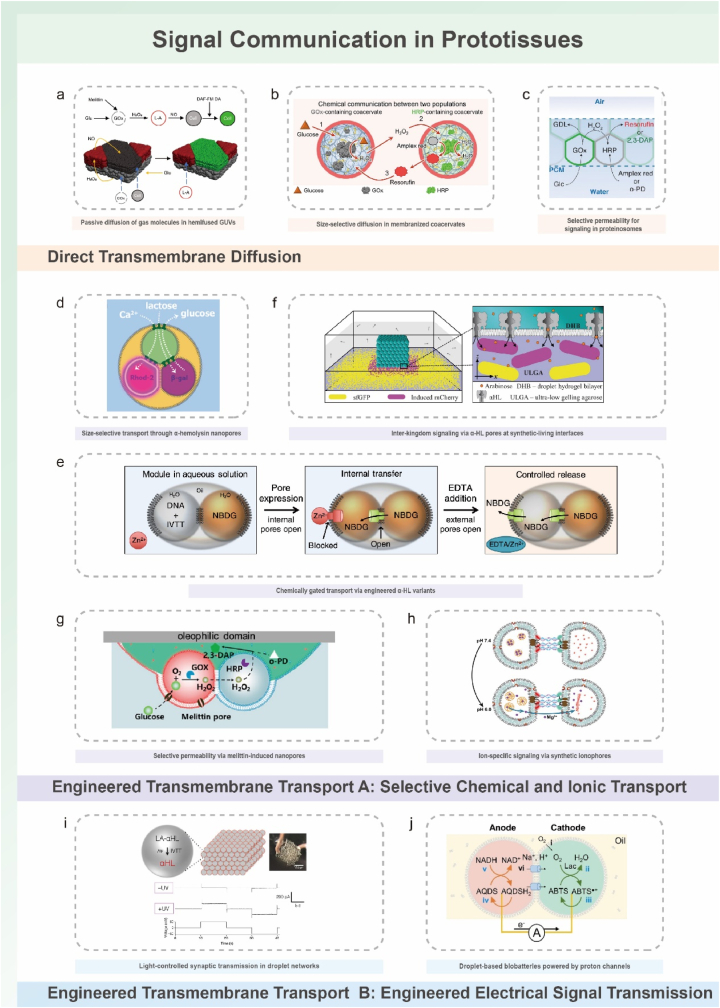
Signal communication pathways in prototissues, spanning passive transmembrane diffusion (a–c), engineered chemical transport (d–h), and engineered electrical signaling (i–j). (a–c) Direct passive transmembrane diffusion. (a) Gas-mediated communication across hemi-fused GUVs, where shared hemifusion diaphragms allow small molecules to passively diffuse between compartments. Reproduced with permission [31]. Copyright 2022, Springer Nature. (b) Size-selective diffusion in membranized coacervates, where molecular transport is governed by inherent permeability of the coacervate–membrane interface. Reproduced with permission [113]. Copyright 2023, American Chemical Society. (c) Selective passive permeability in proteinosomes, enabling small-molecule flux across amphiphilic polymer membranes without engineered channels. Reproduced with permission [62]. Copyright 2021, Wiley-VCH. (d–h) Engineered chemical transmembrane transport via selective biological and synthetic channels. (d) α-Hemolysin (α-HL) nanopores enabling size-selective transport, including lactose, glucose, and fluorogenic substrates. Reproduced with permission [45]. Copyright 2021, American Chemical Society. (e) Chemically gated α-HL variants, where Zn^2+^ binding and EDTA chelation control pore opening/closing and regulate cargo release. Reproduced with permission [36]. Copyright 2019, Springer Nature. (f) Inter-kingdom signaling through α-HL pores inserted at living–synthetic interfaces, enabling α-HL-mediated gene expression activation. Reproduced with permission [42]. Copyright 2025, Wiley-VCH. (g) Melittin-induced nanopores, supporting enzymatic reaction–coupled selective transport (e.g., glucose oxidation and downstream H_2_O_2_ signaling). Reproduced with permission [40]. Copyright 2023, American Chemical Society. (h) Ion-specific communication via synthetic ionophores, enabling proton or ion flux between adjacent protocells and supporting pH-dependent signaling. Reproduced with permission [75]. Copyright 2024, Wiley-VCH. (i–j) Engineered electrical transmembrane signaling. (i) Light-regulated ionic conduction in droplet networks, where UV/visible control of α-HL pore formation modulates transmembrane ion currents, enabling synthetic synaptic-like electrical signaling. Reproduced with permission [43]. Copyright 2016 American Association for the Advancement of Science. (j) Droplet-based bio-batteries, in which coupled redox reactions across anode–cathode droplets generate proton-driven electrical currents for long-range electrical communication. Reproduced with permission [84]. Copyright 2024, Wiley-VCH.

Lipid-based systems illustrate high permeability but limited selectivity. In GUV-based systems, the fluid lipid bilayer permits passive permeation of small, nonpolar molecules. This enables rapid community-wide signaling, as seen in H_2_O_2_ diffusion within hemifused GUV colonies that activates enzymatic cascades [30], or NO diffusion mimicking vascular communication (Fig. 6a) [31]. Similarly, droplet arrays use bilayer permeability to propagate fluorescence pulses via synthetic gene circuits [49]. While effective for broadcast signaling, this mode lacks discrimination, allowing any small molecule to pass.

Coacervate prototissues leverage their intrinsic interfacial properties to introduce size-selective control. Dextran-coated coacervates operate as molecular sieves, excluding macromolecules while permitting small substrates such as H_2_O_2_ and glucose (Fig. 6b) [113]. This inherent selectivity supports compartmentalized enzyme cascades without the need for additional chemical or genetic modification, marking an important step toward functional complexity.

Advanced passive systems incorporate dynamic regulation. Proteinosomes, formed from densely packed protein–polymer nanoparticles, exhibit selective permeability by design [62]. When built from stimuli-responsive polymers (e.g., PNIPAm), permeability becomes tunable: thermo-responsive proteinosomes can transition from an open, diffusive state to a closed state, thereby modulating reaction rates through controlled substrate influx (Fig. 6, Fig. 5h). This provides a direct link between physical stimuli and biochemical output, enabling primitive feedback loops.

In essence, passive diffusion strategies trace a progression from non-selective leakage (lipids) to static molecular filtration (coacervates) and finally to dynamically gated transport (proteinosomes). This evolution enhances the programmability of prototissues, supporting applications in biosensing and adaptive microreactors.

### 5.2 Engineered transmembrane transport

To achieve communication with high spatiotemporal precision and specificity, engineered transmembrane transport strategies are employed. These approaches move beyond broadcast diffusion to enable programmable, directional, and gated exchange of chemical and electrical signals, effectively creating a wired communication network within the prototissue.

#### 5.2.1 Selective chemical and ionic transport

This strategy focuses on controlling the identity and timing of molecular flux through engineered conduits, which can be categorized by their operational principles.

Protein nanopores provide precise control over small-molecule and ionic transport. Engineered protein pores like α-HL provide defined, size-selective channels for small molecules and ions (Fig. 6d) [45]. Their functionality can be further refined with molecular blockers (e.g., cyclodextrins) to prevent crosstalk [34,69] or engineered to be chemically gated (e.g., Zn^2+^-sensitive αHL variants) for on-demand release (Fig. 6e) [36]. This allows for orthogonal reaction control in compartmentalized systems. Furthermore, α-HL pores can bridge synthetic and living systems, mediating chemical communication that triggers gene expression in bacteria (Fig. 6f) [42].

Transient peptide-induced pores enable rapid, burst-type transport events. Peptides such as melittin offer a distinct mode of transport—rapid formation of transient, high-permeability pores upon introduction into the system. These short-lived pores mediate high-flux molecular exchange, enabling fast activation of biochemical pathways such as melittin-enhanced glucose uptake that triggers downstream fluorescent signaling (Fig. 6g) [40]. In pH-regulated protocell communities, melittin also facilitates sucrose and G6P release, thereby supporting metabolic interactions and stepwise NADH production [117]. In contrast to the sustained, size-defined channels formed by α-HL, melittin's short-lived openings offer rapid, episodic transport well suited to dynamic environments.

Ionophores support highly specific ionic signaling without forming large pores. Synthetic ionophores (e.g., ionomycin) facilitate ion-specific transport without forming a macroscopic pore, enabling ionic messengers such as Mg^2+^ to trigger downstream processes—including actin polymerization in receiver protocells—thereby establishing mechanochemical coupling across a prototissue (Fig. 6h) [75].

MOF membranes provide crystalline sieving interfaces with sub-nanometer selectivity. A growing frontier involves using MOFs as crystalline sieving layers on protocell surfaces. When assembled on coacervate droplets, these MOF shells create sub-nanometer, tunable pores capable of precise size- and shape-selective discrimination [87]. This strategy expands interface engineering beyond biological pores, enabling selective transport and capture/release functions with near atomic-level precision.

#### 5.2.2 Ionic and electrical signaling

Leveraging the ion-conducting properties of transmembrane channels enables prototissues to emulate the fastest form of biological communication: electrical signaling.

Switchable ionic circuits arise when ion channels are expressed and activated in a controlled manner. The expression and insertion of ion channels such as α-HL can be controlled spatiotemporally, for example via light-activated gene circuits. This enables prototissue networks to generate switch-like ionic currents, mimicking synaptic transmission and supporting directional signal propagation in 3D-printed architectures (Fig. 6i) [43].

Bioelectronic components based on engineered ion channels enable rectification and current generation. Channels with asymmetric current-voltage characteristics (e.g., arginine-substituted α-HL) create diode-like behavior, enabling biological rectifier circuits capable of elementary computation [38]. Beyond information processing, channels such as gramicidin A enable droplet-based biobatteries that generate sustained electrical currents to power downstream reactions (Fig. 6j) [84].

Taken together, diffusion-based exchange and selective chemical/ionic transport form a complementary communication repertoire, supporting both broad biochemical coupling and finely gated signal transfer. By tuning membrane permeability, pore architecture, channel gating, and interface chemistry—including emerging MOF-based sieving—these systems allow precise control over signal identity, directionality, and temporal dynamics. Taken together, diffusion-based exchange and selective chemical/ionic transport form a complementary communication repertoire, supporting both broad biochemical coupling and finely gated signal transfer. By tuning membrane permeability, pore architecture, channel gating, and interface chemistry—including emerging MOF-based sieving—these systems allow precise control over signal identity, directionality, and temporal dynamics.

## 6 Cutting-edge technologies for prototissue

**Fig. 7:**
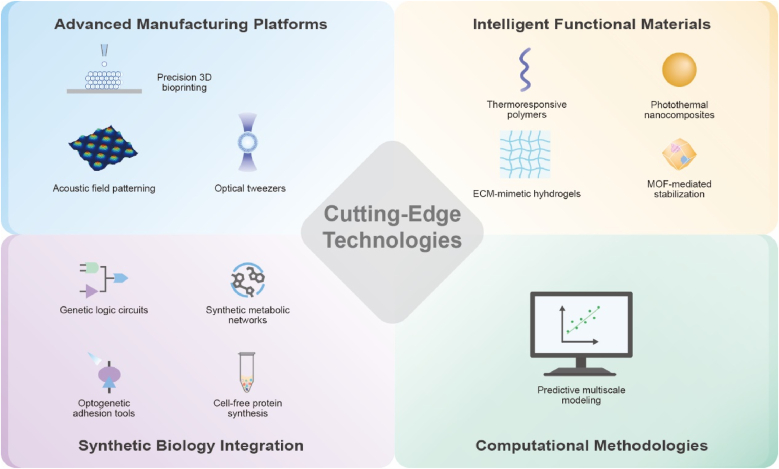
Cutting-edge technologies driving advances in prototissue engineering. Advanced manufacturing tools (3D bioprinting, acoustic field patterning, optical tweezers) enable precise spatial assembly of protocells. Intelligent functional materials (thermoresponsive polymers, photothermal composites, ECM-mimetic hydrogels, MOF-based stabilization) impart adaptive and robust physicochemical properties. Synthetic biology integration (genetic circuits, metabolic pathways, optogenetic adhesion, cell-free synthesis) provides programmable biochemical behaviors. Computational methodologies, particularly predictive multiscale modeling, support rational design of prototissue architectures and functions.

While Sections 2, 3, 4, 5 analyze prototissues through a functional and mechanistic framework (adhesion, spatial organization, stimuli responsiveness, and communication), these capabilities are ultimately enabled by a set of interdisciplinary technologies. Section 6 therefore reorganizes the preceding insights from a technological perspective, highlighting (i) front-line tools that have already transformed prototissue engineering (Fig. 7) and (ii) emerging yet underexploited technologies that may further expand this landscape. This technology-oriented categorization aims to inspire readers from different disciplines to identify how their own toolkits can contribute to future prototissue development.

### 6.1 Advanced manufacturing platforms

Advanced manufacturing technologies provide essential capabilities for establishing hierarchical spatial organization in prototissue systems. Three-dimensional bioprinting remains one of the most advanced strategy, enabling controlled deposition of protocell-laden bioinks to form centimeter-scale architectures with defined spatial heterogeneity [37,42,43,46]. A well-known limitation of printed droplet networks is their dependence on DIBs, which restrict communication with the surrounding aqueous environment; however, solutions such as droplet–hydrogel bilayers (DHBs) have begun to alleviate this constraint by restoring aqueous-phase signal exchange [42].

Complementary field-directed assembly methods—including acoustic [53,[114], [115], [116],^121^], magnetic [31,33,118,124], and optical manipulation [34]—provide non-contact spatial control with micron-level precision and millisecond responsiveness. Magnetic levitation via the Mag-Arch effect, for example, enables rapid, scaffold-free arrangement of GUVs into spatially coded arrays [118], achieving organizational resolution beyond that accessible through printing alone.

Looking ahead, several fabrication modalities remain underexplored yet potentially valuable. High-resolution volumetric printing, microfluidic droplet-on-demand patterning, and automated micromanipulation may offer finer voxel-level encoding or targeted positioning of distinct protocell subpopulations. Although 4D printing has not yet been applied to prototissue construction, its capacity for programmed, stimuli-responsive shape morphing suggests possible utility for future morphogenetic or adaptive architectures. These prospects remain speculative but illustrate rational directions for extending current manufacturing capabilities.

Overall, advanced manufacturing platforms establish the structural foundation of prototissue engineering by enabling scalable, precise spatial organization. Continued integration of emerging fabrication technologies—approached cautiously and grounded in mechanistic understanding—may support increasingly complex and reconfigurable prototissue architectures.

### 6.2 Intelligent functional materials

Intelligent functional materials provide the mechanistic basis for many of the dynamic, adaptive, and mechanoresponsive behaviors exhibited by prototissues, extending their functionality far beyond static assemblies. Within existing studies, thermoresponsive PNIPAm-based matrices have been widely employed to achieve reversible shape morphing and volumetric transitions, enabling prototissue contraction, folding, or relaxation under mild temperature changes and thereby supporting the mechanically coupled behaviors [48,60,64]. AuNP-enabled photothermal systems have similarly been leveraged to induce localized heating, deformation, and directional actuation [64], while magnetic nanoparticles and ferromagnetic micromaterials have allowed remote, non-contact manipulation, rotational control, and cargo transport within cellular and noncellular PTMs [42,46,48,57,58]. In parallel, ECM-mimetic hydrogels [60,[80], [81], [82]], mineralizing polymer networks [82], and coacervate-based membranes [83] contribute to structural fidelity and mechanochemical feedback by introducing stiffness gradients, self-thickening fusion interfaces, or dynamic mineral shells that can reversibly reorganize under Fe^2+^/Fe^3+^ switching. Together these examples illustrate how intelligent materials collectively underpin shape-programmability, mechanical reinforcement, and responsive behaviors across many prototissue systems.

These material platforms, already enabling diverse modes of actuation and structural adaptation, may also be further extended or combined to support broader functional possibilities in future prototissue systems.

### 6.3 Synthetic biology integration

Synthetic biology introduces molecular-level programmability that increasingly connects prototissue behavior to genetically encoded adhesion, signaling, and communication processes, providing a complementary layer to the material- and assembly-driven strategies discussed in earlier sections. Existing prototissue studies have demonstrated how engineered membrane proteins, such as K3/E3-modified α-HL variants, enable controlled electrostatic adhesion and selective protocell coupling, while optogenetic pairs including iLID–Nano [[69], [70], [71]] or PhyB–PIF6 [70] mediate reversible, light-dependent aggregation, disassembly, and Ca^2+^ signaling wave propagation within multi-member communities. Engineered nanopores and synthetic channels—including α-HL, αHL-4H, and Zn^2+^-gated or chemically modulated variants [34,36,44,46,49,51,59,71]—further support programmable molecule exchange, electrical conduction, and coordinated activation cycles, often reinforced by cell-free expression systems [36,41,43,44,46,119] that allow on-demand production of membrane components or signaling proteins. These capabilities have been employed to implement hierarchical communication, predator–prey interactions, multi-color logic gating, and spatiotemporally controlled adhesion behaviors described in Section 5, collectively positioning synthetic biology as a route toward increasingly autonomous and life-like prototissue systems. Additionally, DNA nanotechnology—comprising cholesterol-anchored complementary single-stranded DNA, DNA-origami structures, and DSD reactions—delivers dynamic, stimulus-responsive reconfiguration with nanoscale precision, enabling geometric control over scalable architectures [[74], [75], [76]]. When coupled with optogenetic tools, DSD circuits further establish logic-gated communication pathways that may expand collective behavioral complexity [70]. Moreover, synthetic metabolic networks (e.g., mitochondrial ATP-driven contraction, photo-triggered enzymatic cascades) integrate endogenous energy transduction with spatial organization [32,81]. These approaches collectively exemplify how gene-encoded precision and modular design support the development of environmentally responsive prototissue systems with implications for biomedical and bionic applications. As these synthetic-biology modules continue to interface with protocellular assemblies, their programmable principles may likewise be leveraged to enrich the range of coordinated behaviors achievable in prototissues.

### 6.4 Computational methodologies

Computational approaches offer theoretical frameworks for analyzing and predicting complex behaviors in prototissue systems, thereby bridging molecular-scale mechanisms with collective dynamics. The "cellular Turing test" concept, inspired by computational principles, provides a metric to evaluate the fidelity of communication between artificial and natural cells by quantifying the successful activation of quorum-sensing pathways [126]. Multiscale modeling—combining reaction-diffusion equations with stochastic processes—deciphers spatiotemporal signal propagation: effective diffusion coefficients predict transmembrane permeation rates of signaling molecules (e.g., arabinose, C6-HSL) in lipid bilayer networks [39], while Hill-function-coupled promoter models replicate observed gene expression patterns [49]. For spatially structured systems, continuum models simulate signal pulse attenuation in 1D/2D protocell arrays, revealing geometric dependencies of communication range. Stochastic analyses further identify noise sources (e.g., stochastic pore insertion) driving phenotypic variability in synthetic cell populations [80]. Complementarily, quantitative biophysical models—such as radial distribution frameworks for light-activated iLID-SspB dimerization—precisely map interfacial signaling kinetics, enabling comparative evaluation of external versus endogenous activation efficiency (e.g., 30 % for luciferase-based systems versus near-complete external activation) [127]. These models guide experimental parameter optimization and rationalize performance limitations. While computational tools accelerate the transition from qualitative construction toward quantitative engineering, their predictive power remains constrained by simplifications in boundary conditions and incomplete incorporation of microenvironmental heterogeneity.

Looking forward, the field stands to be transformed by artificial intelligence (AI) and machine learning (ML). Beyond current modeling, these tools offer a pathway to inverse design: given a target function (e.g., a specific drug release profile or locomotion pattern), AI models could identify the optimal combination of protocell composition and spatial architecture to achieve it. Furthermore, generative models may accelerate the discovery of novel building blocks, such as designing custom adhesion proteins or copolymer membranes with programmed properties. The integration of these predictive computational tools with high-throughput experimental platforms will ultimately establish a closed-loop design cycle, positioning computation as a co-pilot in the intelligent engineering of adaptive prototissues.

In sum, the integration of precision manufacturing, intelligent materials, synthetic biology, and computational methodologies demonstrates transformative potential in advancing prototissue engineering. The future convergence of these domains will accelerate the transition from proof-of-concept prototypes to clinically relevant biohybrid systems and intelligent bionic devices.

## 7 Emerging applications and functional opportunities

**Fig. 8:**
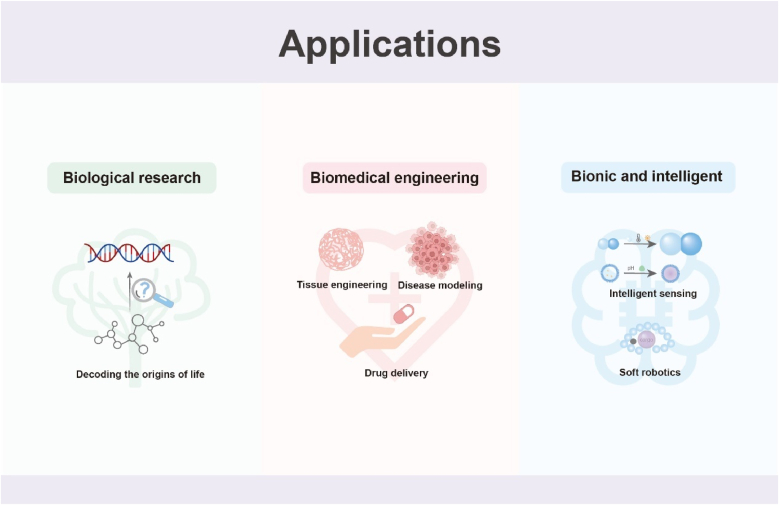
Potential application directions of prototissues. Prototissues provide simplified multicellular constructs that may facilitate future biological research, such as exploring early cellular organization and chemical scenarios relevant to the origins of life. In biomedical engineering, prototissues hold potential as model systems for tissue construction, disease simulation, and programmable drug delivery strategies. In bionic and intelligent technologies, prototissues offer prospective avenues for developing responsive sensing modules and soft robotic components inspired by biological functions.

Prototissues offer emerging opportunities across scientific and technological domains by providing programmable architectures with finely tunable adhesion, communication, and stimuli responsiveness. Although still at a developmental stage, these systems enable controlled perturbations and engineered interactions that are difficult to isolate in living tissues or achieve with conventional biomaterials. Their potential lies not in replicating full biological complexity, but in offering reductionist yet reconfigurable platforms to probe biological principles and explore early concepts for translational strategies (Fig. 8).

### 7.1 Model systems for understanding complex biological phenomena

Prototissues function as experimental models for dissecting how spatial organization, transport selectivity, and collective communication contribute to emergent behavior. Their composition and interaction rules can be independently tuned, allowing researchers to reconstruct minimal multicellular scenarios and evaluate how geometry or resource distribution influences cooperative catalysis or metabolic stability. Such systems enable quantitative tests of hypotheses regarding early multicellularity and abiotic-to-biotic transitions, where controlled transport pathways and adhesion modes can be systematically varied—conditions difficult to manipulate in natural systems [13].

The ability to precisely engineer spatial organization provides a direct pathway to uncover how geometric structure governs tissue-level functionality. This mechanistic link is exemplified by studies where defined spatial configurations directly dictate emergent functions. For instance, the spatial arrangement of different protocell populations, achieved via magnetic coding, can suppress the dynamic feedback essential for community-level pH oscillations, demonstrating that geometric organization is a fundamental parameter governing collective behavior [117]. In another approach, concentric layering of enzyme-rich protocells creates a reaction-diffusion network that performs logic-gated signal processing, precisely regulating the output of bioactive nitric oxide in the central lumen for anticoagulation [80]. Furthermore, geometric control over droplet packing (e.g., into hexagonal close-packed lattices) enables the fabrication of single-droplet-wide conductive pathways within 3D-printed tissues, functionally mimicking specialized structures like neuronal tracts [37]. These examples collectively demonstrate that spatial architecture is not merely a scaffold but a direct influence of higher-order functions, by controlling molecular diffusion paths and reaction-diffusion dynamics.

Embedding prototissues within hydrogels or responsive matrices further allows investigation of microenvironment-mediated regulation, including how osmotic, mechanical, or thermal cues guide reaction–diffusion patterns and symmetry breaking [81].These engineered assemblies provide tractable testbeds to explore mechanochemical principles underlying morphogenesis and tissue-level coordination. By bridging materials science and synthetic biology, prototissues offer a modular and perturbation-friendly approach for studying how higher-order biological functions arise from simple interaction rules.

### 7.2 Prototissue-mediated control of biological microenvironments

In biomedical contexts, prototissues present a promising strategy for modulating local biochemical and biophysical microenvironments. Their capacity to couple spatial organization with responsive molecular transport offers a path—still largely conceptual—for influencing cellular behavior in regenerative medicine. For example, prototissue-hydrogel hybrids are uniquely positioned to provide dynamic, time-dependent cues that guide complex processes like tissue maturation and vascularization, moving beyond the static support offered by conventional scaffolds [81]. For instance, a thermoresponsive hydrogel–prototissue system can be designed to evolve its mechanical properties *in situ*. As the temperature shifts slightly around physiological conditions, the hydrogel matrix can undergo a phase transition, progressively increasing its stiffness to mimic the natural hardening of the extracellular matrix during tissue development. Simultaneously, the embedded prototissues can respond to this mechanical change or to external triggers (e.g., light, specific metabolites) by precisely releasing morphogens, such as VEGF. This creates a feedback loop where the evolving matrix mechanics and the spatiotemporally controlled release of biochemical signals from the prototissues work in concert to first promote stem cell differentiation and subsequently guide the assembly of nascent endothelial cells into capillary-like structures.

Similarly, the modularity of protocellular compartments enables targeted and stimulus-responsive therapeutic concepts, including aptamer-mediated localization or controlled co-delivery of synergistic agents. While these strategies remain exploratory, they highlight how programmable permeability and adhesion may support microenvironment-driven drug action.

Prototissues also offer value as disease modeling tools, particularly where dynamic control of gradients, barriers, or host–pathogen interactions is essential. Acoustic trapping platforms [30] and hybrid DIB systems [39] have been used to interrogate antimicrobial diffusion and real-time microbial responses, demonstrating how engineered assemblies can complement traditional *in vitro* models by capturing aspects of spatial heterogeneity or barrier function.

### 7.3 Programmable sensing, computation, and actuation

Beyond biological research and early biomedical concepts, prototissues serve as test platforms for chemical sensing, distributed computation, and soft actuation. Spatially organized droplet or vesicle arrays can amplify weak biochemical cues and implement logic-like responses through cascaded reactions or gated transport, improving adaptability relative to static biosensors [40,49].These architectures provide insight into how spatial partitioning and controlled communication can support information processing.

Prototissue-based soft robotic behaviors represent another emerging direction. Systems exhibiting osmotic–thermal coordination or enzyme-mediated predator–prey-like responses demonstrate how mechanochemical communication can drive adaptive motion or reconfiguration [81].While far from practical devices, these examples underscore the value of prototissues as concept platforms for understanding how sensing, computation, and actuation may be integrated within soft-matter architectures.

Overall, prototissues function less as application-ready materials and more as flexible exploratory platforms that reveal how programmed adhesion, transport, and communication can generate complex behaviors. Their strength lies in offering controllable, reductionist environments for probing biological principles and for prototyping microenvironment-responsive therapeutic or sensing strategies. As design tools for emerging technologies, they provide a foundation for future advances in model biological systems, adaptive biomaterials, and soft-matter devices capable of integrating sensing, computation, and actuation in a unified manner.

## 8 Challenges

Despite significant advances in prototissue engineering, several fundamental challenges persist across hierarchical design scales—from molecular-level adhesion to macroscale functional integration. These interconnected limitations critically constrain the emulation of native tissue complexity and its potential for translation into real-world biomedical applications. To systematically contextualize these bottlenecks, Table 4 summarizes the core gaps between current prototissue capabilities and the sophisticated properties of native tissues. This section subsequently examines the four pivotal bottlenecks highlighted in the table: biomimetic fidelity in inter-protocell adhesion, scalability-functionality trade-offs, the primitive nature of collective behaviors, and barriers to clinical biointegration, while highlighting their cross-cutting interdependencies.

**Table 4:** Summary of key challenges in prototissue engineering: Current limitations versus prospective biomimetic visions.

Dimension	Native tissue benchmark	Current prototissue limitation	Core gap	Prospective future pathway
Adhesion & connectivity	Dynamic force-responsive adhesion (e.g., cadherins), reversible connectivity, metabolic regulation	Static or crudely reversible adhesion (e.g., pH or ionic-strength dependence), lack of mechanosensitivity	Absence of life-like dynamism and feedback	Engineered biomimetic adhesives (e.g., connexin-mimetic proteins, force-sensitive DNA origami)
Spatial architecture & scalability	Hierarchical vascularized networks enabling mass transport	Scale–resolution trade-offs, absence of internal perfusion	Inability to reconcile size with functional integrity	Modular assembly with synthetic vasculature (e.g., 3D-printed perfusable channels)
Collective behaviors & signaling	Complex adaptive behaviors (e.g., chemotaxis, self-healing), multimodal communication (chemical, electrical)	Primitive isotropic responses (e.g., swelling), short-range passive diffusion–dominated signaling	Limited behavioral repertoire and communication range	Integration of synthetic gene circuits for logic-gated responses and long-range electrical signaling
Biointegration & compatibility	Seamless host integration, immune compatibility, metabolic synchronization	Immune activation, mechanical mismatch, metabolic dysregulation at host interface	Hostile host-prototissue interface	“Immuno-stealth” materials, gradient scaffolds matching tissue stiffness, embedded metabolic synchronizers

### 8.1 Biomimetic deficits in inter-protocell adhesion

Current approaches for inter-protocell adhesion primarily depend on abiotic interactions (e.g., DIBs, electrostatic crosslinking, covalent bonds), especially in lipid-based systems like GUVs. While these strategies ensure structural cohesion, they critically lack dynamic force responsiveness and biocompatibility, failing to emulate biological mechanisms such as cadherin mechanotransduction. The underdevelopment of biomolecule-mediated adhesion (e.g., DNA hybridization with suboptimal binding affinity, light-dependent tools with poor tissue penetrance) further restricts adaptive signaling in prototissue systems. Crucially, deficient ECM-mimetic scaffolds impede effective stress dissipation, mechanochemical coordination, and dynamic intercellular communication, directly impairing advanced tissue behaviors such as force transmission and collective motion. Overcoming these limitations will require developing biomolecule-mediated adhesion systems with enhanced affinity, tunability, and compatibility with surrounding tissue environments.

### 8.2 Scalability-functionality trade-offs in macroscale engineering

The construction of centimeter-scale prototissues faces inherent trade-offs between macroscopic expansion and spatial resolution. While template-guided assembly (e.g., floating molds, hydrogel scaffolds) enables large-scale prototissue construction, it compromises the control over signal directionality and heterogeneous protocell positioning. Conversely, 3D printing enables geometric complexity but impedes aqueous signaling by encapsulating DIBs in hydrophobic phases, severely restricting transmembrane diffusion of water-soluble molecules like Ca^2+^ and ATP. Moreover, the lack of vascularization-mimetic networks at larger scales exacerbates delays in signal propagation and reduces collective coordination, mimicking the challenges in natural tissue engineering. These scalability constraints hinder functional fidelity and significantly challenge biointegration, as prototissues struggle with nutrient/waste exchange and mechanochemical synchronization with host tissues. To address this issue, it will be essential to explore vascularization strategies such as microfluidic-based perfusion and self-assembled vascular networks to improve large-scale functionality.

### 8.3 Primitive collective behaviors and signaling limitations

Prototissues currently exhibit primitive collective behaviors that are constrained by oversimplified mechanochemical responses, such as isotropic swelling or shrinkage driven by osmotic or thermal stimuli. These systems fail to replicate more advanced behaviors, including chemotaxis or self-healing. Communication in prototissues still relies heavily on passive small-molecule diffusion, limiting the coordination of large-scale behaviors to submillimeter scales. Although engineered pores allow for limited spatiotemporal control, they are insufficient for enabling feedback-regulated oscillations or more complex signaling such as electrical impulses or photonic signaling. Moreover, current systems suffer from inefficient mechano-signaling coupling. This inefficiency stems from fundamental limitations in energy conversion and signal transduction fidelity within synthetic materials, which fail to match the highly organized and enzyme-driven processes of the cytoskeleton in living cells. Consequently, the resulting force outputs are orders of magnitude lower than those of biological systems, undermining environmental navigation and reducing the ability to integrate complex, adaptive behaviors. Future efforts must focus on multimodal signaling capabilities (e.g., coupling chemical, electrical, and mechanical signals) and the integration of self-healing and chemotactic behaviors within prototissue systems.

### 8.4 Biocompatibility and biointegration barriers

The biomedical application of prototissues is hindered by significant challenges related to biocompatibility and biointegration. Non-physiological components—such as residual heavy metal catalysts from click chemistry or adhesion strategies requiring high ionic strength—can provoke chronic inflammation and immune rejection. Furthermore, mismatches in mechanical properties—such as the excessive stiffness of synthetic matrices versus the softness of biological tissues—cause interfacial stress and disrupt coordinated biological processes. Additionally, dysynchronous metabolic rhythms between synthetic and native tissues contribute to mismatched integration, causing structural delamination at graft-host junctions and functional desynchronization *in vivo*. These issues highlight the need for materials that not only possess suitable mechanical properties but also demonstrate metabolic compatibility. The development of immune-stealth strategies and gradient scaffolds matching tissue-specific Young's moduli could improve integration and reduce rejection. Despite these limitations, prototissues have demonstrated their potential in mimicking native tissue architectures *in vitro*, and resolving these challenges will unlock their therapeutic potential.

In conclusion, these challenges form a self-reinforcing network of constraints. Biomimetic adhesion deficits directly limit the complexity of behaviors, while scalability limitations amplify biointegration challenges. Resolving these intertwined issues requires co-engineering strategies that integrate dynamic bioadhesives, vascularized architectures, neuromorphic signaling systems, and adaptive regulatory mechanisms. Addressing these bottlenecks will unlock the potential of prototissues as programmable platforms for decoding biological complexity and enabling transformative biomedical applications.

## 9 Prospects

**Fig. 9:**
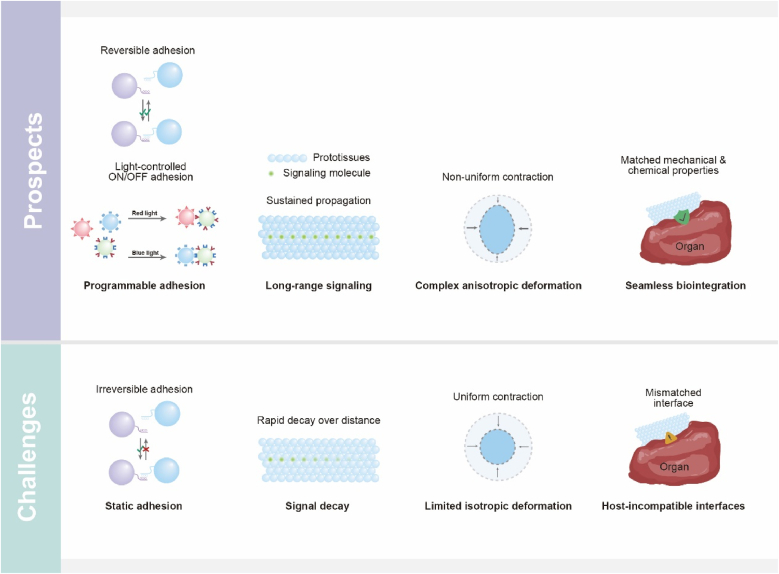
Challenges in current prototissue systems (bottom) and corresponding prospective properties envisioned for future designs (top). Present limitations include static and irreversible adhesion, rapid signal decay over distance, isotropic and weakly coordinated collective responses, and host-incompatible interfaces that hinder integration with biological tissues. In contrast, future prototissues are anticipated to exhibit programmable (e.g., light-controlled ON/OFF linkages) and reversible adhesion, sustained long-range chemical communication, complex and anisotropic motility, and seamless biointegration with host environments. These property-level advancements highlight how overcoming current bottlenecks may enable next-generation prototissues with enhanced adaptability, coordination, and biomedical compatibility.

The evolution of prototissues demands coordinated advancements across multiple domains to address the current challenges and realize future prospects (Fig. 9). Progress in these areas must be both gradual and systematic, overcoming the bottlenecks identified in Section 8.

### 9.1 Biomimetic adhesion and communication

Future advancements should focus on integrating stimuli-responsive, connexin-mimetic proteins to establish on-demand inter-protocell connectivity, alongside DNA-origami interfaces functionalized with signaling molecules to form dual-purpose junctions that synchronize both adhesion and communication. This approach will enable more flexible, responsive systems capable of mimicking the dynamic adhesion seen in biological tissues. Additionally, ECM-mimetic matrices should incorporate tunable viscoelasticity and protease-sensitive motifs to better replicate natural mechanotransduction, facilitating the emergence of collective behaviors in prototissue systems.

### 9.2 Overcoming scalability limitations

Addressing scalability challenges will require the modular assembly of pre-programmed protocell units, which can be interconnected by synthetic vascular networks. One promising solution is microfluidic 3D printing of perfusable hydrogel channels to sustain metabolite exchange and nutrient flow in centimeter-scale prototissues, while also enabling dynamic regulation of reaction-diffusion signals through embedded enzyme cascades. This strategy will decouple spatial resolution from size, enabling large-scale prototissue constructs with functional integrity.

### 9.3 Enhancing collective behaviors

To achieve more complex collective functions, material innovations such as strain-adaptive block copolymer membranes should be explored to enable anisotropic responses, such as peristalsis. Additionally, synthetic gene circuits in DNA-engineered protocells could convert chemical gradients into electrical pulses through engineered ion channels (e.g., rectifying α-HL variants). The integration of photonic signaling will further facilitate the development of multimodal networks for chemotaxis, self-healing, and other advanced behaviors.

### 9.4 Clinical translation and biointegration

Successful clinical translation of prototissues will hinge on the development of host-adaptive strategies. For instance, immuno-stealth materials (e.g., CD47-mimetic polymer conjugates) could evade immune surveillance, while gradient scaffolds designed to match the tissue-specific Young's moduli (ranging from 1 kPa to MPa) will ensure mechanical compatibility. Additionally, embedding metabolic synchronizers (e.g., NAD+/NADH sensors) within protocells could help align synthetic-native tissue rhythms, requiring validation in dynamic *in vivo* models to ensure functional integration.

### 9.5 Accelerating design with AI and computational tools

Finally, the application of artificial intelligence-driven design will significantly accelerate prototissue development. Machine learning models could predict immune responses, optimizing biocompatibility, while tools like AlphaFold could enable atomic-precision design of force-sensitive adhesion proteins. Furthermore, generative neural networks could optimize protocell compositions for targeted functionalities such as drug release, tested and validated through closed-loop microfluidic platforms.

Collectively, these innovations position prototissues as adaptive biological proxies that bridge molecular precision with tissue-level functionality, marking a transformative shift in bioengineering and biomedicine.

## 10 Conclusions

Prototissues—engineered assemblies of interacting protocells—demonstrate how compartmentalization, programmable adhesion, and spatial organization can be integrated to generate collective behaviors beyond the capabilities of individual synthetic units. Through the dual-dimensional framework synthesized in this review, molecular-level adhesion strategies are shown to define interfacial connectivity, while meso-to macroscale spatial programming governs emergent architecture and function. Together, these design axes outline generalizable principles for constructing soft, tissue-like materials with life-inspired properties.

Beyond consolidating recent developments, this review contributes by translating a diverse and technically fragmented landscape into a coherent design perspective. By mapping materials, adhesion chemistries, spatial programming strategies, and communication modalities onto a structured organizational logic, the review clarifies how specific construction decisions drive emergent functionalities. This integrative view provides an accessible conceptual entry point for newcomers while offering a strategic foundation for rational prototissue engineering.

Recent advances reveal a clear shift from assembling isolated protocells to developing multi-component systems capable of information processing, environmental responsiveness, and cooperative function. The incorporation of stimuli-responsive materials, synthetic biological modules, and engineered communication interfaces has enabled adaptive mechanics, mechanochemical coupling, and coordinated signaling. These capabilities position prototissues as powerful platforms for both interrogating fundamental principles of multicellular organization and enabling applications in biomedicine, biosensing, and soft robotics.

Despite this progress, critical challenges remain. Adhesion mechanisms still lack the dynamicity and mechanosensitivity characteristic of natural cell–cell interfaces; scaling architectures without sacrificing spatial resolution or signal fidelity is difficult; collective behaviors remain largely limited to simple, isotropic responses; and materials incompatibilities constrain biological integration. These limitations are interdependent, underscoring the need for co-engineered solutions that unify materials chemistry, synthetic biology, and microscale fabrication.

Looking ahead, meaningful advances will likely arise from interdisciplinary convergence. Integrating dynamic bioadhesives with architected, perfusable materials; coupling synthetic gene circuits with programmable mechanical elements; and applying computational tools for predictive design may collectively expand the functional landscape of prototissues. As these systems evolve, they may serve dual roles: as reductionist platforms for dissecting principles of biological organization and as adaptive engineered constructs with controllable, life-like behaviors for technological and biomedical applications.

Rather than replicating natural tissues, prototissues offer a complementary design pathway—one that leverages modularity, programmability, and synthetic flexibility to explore new forms of collective organization. Continued progress holds promise for deepening our understanding of biological complexity while establishing a versatile foundation for future bioinspired materials and systems.

## CRediT authorship contribution statement

**Ziqi Liu:** Writing – original draft, Visualization, Validation, Methodology, Investigation, Formal analysis. **Yiming Wang:** Methodology, Investigation. **Wei Pei:** Methodology, Investigation. **Yi-Xin Huo:** Supervision, Project administration. **Yuan Lu:** Writing – review & editing, Supervision, Project administration, Funding acquisition, Conceptualization.

## Ethics approval and consent to participate

This manuscript is a literature review work, and thus no *in vivo* evaluations on animal model or clinical trials were performed in this scope. Thereby, our work does not fall into the incidence of ethical approvals and patient consents.

## Declaration of competing interest

The authors declare that they have no known competing financial interests or personal relationships that could have appeared to influence the work reported in this paper.
